# Evidences from a Systematic Review and Meta-Analysis Unveil the Role of MiRNA Polymorphisms in the Predisposition to Female Neoplasms

**DOI:** 10.3390/ijms20205088

**Published:** 2019-10-14

**Authors:** Milad Bastami, Jalal Choupani, Zahra Saadatian, Sepideh Zununi Vahed, Elaheh Ouladsahebmadarek, Yasser Mansoori, Abdolreza Daraei, Hossein Samadi Kafil, Bahman Yousefi, Mahdi Mahdipour, Andrea Masotti, Ziba Nariman-Saleh-Fam

**Affiliations:** 1 Department of Medical Genetics, Faculty of Medicine, Tabriz University of Medical Sciences, Tabriz 5166614756, Iran; mi.bastami@live.com; 2 Immunology Research Center, Tabriz University of Medical Sciences, Tabriz 5166614756, Iran; chupani.genetic@gmail.com (J.C.); kafilhs@tbzmed.ac.ir (H.S.K.); 3 Department of Basic Sciences, Faculty of Medicine, Gonabad University of Medical Sciences, Gonabad 9691793718, Iran; z.saadatian@yahoo.com; 4 Kidney Research Center, Tabriz University of Medical Sciences, Tabriz 5166614756, Iran; sepide.zununi@gmail.com; 5 Women′s Reproductive Health Research Center, Tabriz University of Medical Sciences, Tabriz 5138663134, Iran; elmadarek33@gmail.com; 6 Noncommunicable Diseases Research Center, Fasa University of Medical Sciences, Fasa 7461686688, Iran; fums.mansoori@gmail.com; 7 Department of Genetics, Faculty of Medicine, Babol University of Medical Sciences, Babol 4617647745, Iran; a.daraei@mubabol.ac.ir; 8 Drug Applied Research Center, Tabriz University of Medical Sciences, Tabriz 5165665811, Iran; bahmanusefi@gmail.com; 9 Stem Cell Research Center, Tabriz University of Medical Sciences, Tabriz 5166614756, Iran; mahdi.mahdipour@gmail.com; 10 Department of Reproductive Biology, Faculty of Advanced Medical Sciences, Tabriz University of Medical Sciences, Tabriz 5166614766, Iran; 11 Research Laboratories, Bambino Gesù Children′s Hospital-IRCCS, Rome 00146, Italy

**Keywords:** microRNA, polymorphism, breast neoplasm, female neoplasm, susceptibility, cancer

## Abstract

Breast (BCa) and gynecological (GCa) cancers constitute a group of female neoplasms that has a worldwide significant contribution to cancer morbidity and mortality. Evidence suggests that polymorphisms influencing miRNA function can provide useful information towards predicting the risk of female neoplasms. Inconsistent findings in the literature should be detected and resolved to facilitate the genetic screening of miRNA polymorphisms, even during childhood or adolescence, and their use as predictors of future malignancies. This study represents a comprehensive systematic review and meta-analysis of the association between miRNA polymorphisms and the risk of female neoplasms. Meta-analysis was performed by pooling odds-ratios (ORs) and generalized ORs while using a random-effects model for 15 miRNA polymorphisms. The results suggest that miR-146a rs2910164 is implicated in the susceptibility to GCa. Moreover, miR-196a2 rs11614913-T had a moderate protective effect against female neoplasms, especially GCa, in Asians but not in Caucasians. MiR-27a rs895819-G might pose a protective effect against BCa among Caucasians. MiR-499 rs3746444-C may slightly increase the risk of female neoplasms, especially BCa. MiR-124 rs531564-G may be associated with a lower risk of female neoplasms. The current evidences do not support the association of the remaining polymorphisms and the risk of female neoplasms.

## 1. Introduction

Breast (BCa) and gynecological (GCa) cancers constitute a group of female neoplasms (ICD-10: C50, C51–58) that has significant contribution to cancer morbidity and mortality worldwide. Despite advances in the screening and treatment of cancer [1,2,3], BCa is still the most frequent malignancy, accounting for more than 24% of all cases, and the leading cause of cancer death among females globally [4]. Gynecological cancers (GCa) or neoplasms of the female genital organs (ICD-10: C51–C58) include cervical, ovarian, endometrial, vaginal, vulvar, and fallopian tube cancers, among which cervical cancer (CCa) and ovarian cancer (OCa) are among the 10 most common cancers in females [4]. Tumorigenesis of these cancers is an intricate process that is influenced by both environmental and genetic factors, ranging from carcinogens and reproductive factors to genetic components [5]. Accumulating evidences supports a role for genetic predisposition factors in the epidemiology of BCa and GCa. Recently, extensive research stressed the role of noncoding RNAs, especially microRNAs (miRNAs), in carcinogenesis and susceptibility to several cancers, including BCa and GCa [6,7,8].

miRNAs are short non-coding RNAs that are involved in negatively regulating the expression of most protein-coding genes in the post-transcriptional level [9]. They are engaged in complex networks that are responsible for tight regulation of important cellular processes that are often altered during carcinogenesis [10]. The utility of miRNAs as early detectors of different cancers is under active research. Increasing evidence suggest that single nucleotide polymorphisms (SNPs) influencing miRNA function (i.e., miRNA polymorphisms) can provide useful information to predict the risk of female neoplasms [11,12,13,14,15]. Recent studies reported several miRNA-related polymorphisms that can play pivotal roles in the prediction of BCa and GCa risk development and emphasized their utility as reliable genetic markers for predicting potential cancer risk [11,13,14]. Therefore, the genetic screening for miRNA polymorphisms, even during childhood or adolescence, holds great promise and offers the opportunity to better define the genetic risk to female neoplasms by exploiting these early predictors of future malignancies. However, inconsistent findings found in the literature should be detected and resolved to facilitate the use of miRNA polymorphisms in diagnostics as predictors of future malignancies. 

This study represents a comprehensive systematic review and meta-analysis of the risk of female neoplasms that are associated with miRNA polymorphisms. We were interested in finding: which miRNA polymorphisms are hypothesized to modify the risk of female neoplasms, including breast and gynecological cancers in the literature, whether there are sufficient data to draw robust conclusions regarding the role of miRNA polymorphisms in the susceptibility to different female neoplasms, whether miRNA polymorphisms pose ethnic-specific effects in the susceptibility to female neoplasms. 

## 2. Results and Discussion

### 2.1. Study Characteristics

**Figure 1:**
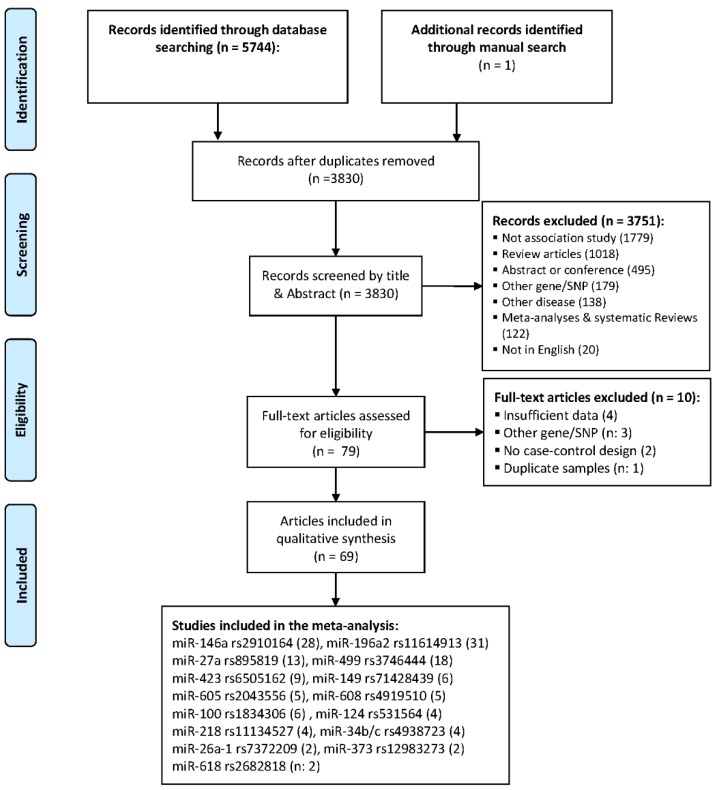
The process of study selection.

Figure 1 shows the process of identifying eligible studies. A total of 5745 records were identified through a literature search. After removing duplicate records, titles and abstracts of the remaining 3830 records were screened and 3751 records were excluded for the following reasons: not a genetic association study (*n*: 1779), review articles (*n*: 1018), abstracts or conference papers (*n*: 495), studying other genes or polymorphisms (*n*: 179), studying other diseases (*n*: 138), systematic reviews or meta-analyses (*n*: 122), and not written in English (*n*: 20). The full texts of the remaining 79 records were evaluated and 10 articles were excluded for the following reasons: insufficient reported data (*n*: 4) [16,17,18,19], studying other genes or polymorphisms (*n*: 3) [20,21,22], not a case-control design (*n*: 2) [23,24], and a duplicate study (*n*: 1) [25]. Finally, a total of 69 articles remained [26,27,28,29,30,31,32,33,34,35,36,37,38,39,40,41,42,43,44,45,46,47,48,49,50,51,52,53,54,55,56,57,58,59,60,61,62,63,64,65,66,67,68,69,70,71,72,73,74,75,76,77,78,79,80,81,82,83,84,85,86,87,88,89,90,91,92,93]. Taken together, the association of 65 miRNA polymorphisms with the risk of female neoplasms (either BCa or GCa) was evaluated in these articles. Meta-analyses were performed for 15 miRNA polymorphisms, for which the number of studies per SNP was more than one. Table 1 summarizes the main characteristics of studies that were included in the final meta-analysis. 

The meta-analyses were performed for the remaining 15 miRNA polymorphisms. These include: miR-146a rs2910164 (28 studies, 11,071 cases and 12,312 controls), miR-196a2 rs11614913 (31 studies, 11034 cases, and 12955 controls), miR-27a rs895819 (13 studies, 6743 cases, and 8461 controls), miR-499 rs3746444 (18 studies, 7627 cases, and 9489 controls), miR-423 rs6505162 (nine studies, 3505 cases, and 4273 controls), miR-149 rs2292832 (six studies, 2211 cases, and 2422 controls), miR-605 rs2043556 (five studies, 2706 cases, and 3804 controls), miR-608 rs4919510 (five studies, 2115 cases, and 3189 controls), miR-100 rs1834306 (six studies, 1969 cases, and 2192 controls), miR-124 rs531564 (four studies, 1213 cases, and 1312 controls), miR-218 rs11134527 (four studies, 3134 cases, and 2966 controls), miR-34b/c rs4938723 (four studies, 2536 cases, and 2535 controls), miR-26a-1 rs7372209 (two studies, 295 cases, and 608 controls, miR-373 rs12983273 (two studies, 955 cases, and 920 controls), and miR-618 rs2682818 (two studies, 684 cases, and 1039 controls).

### 2.2. miR-146a rs2910164 and the Risk of Female Neoplasms

**Figure 2:**
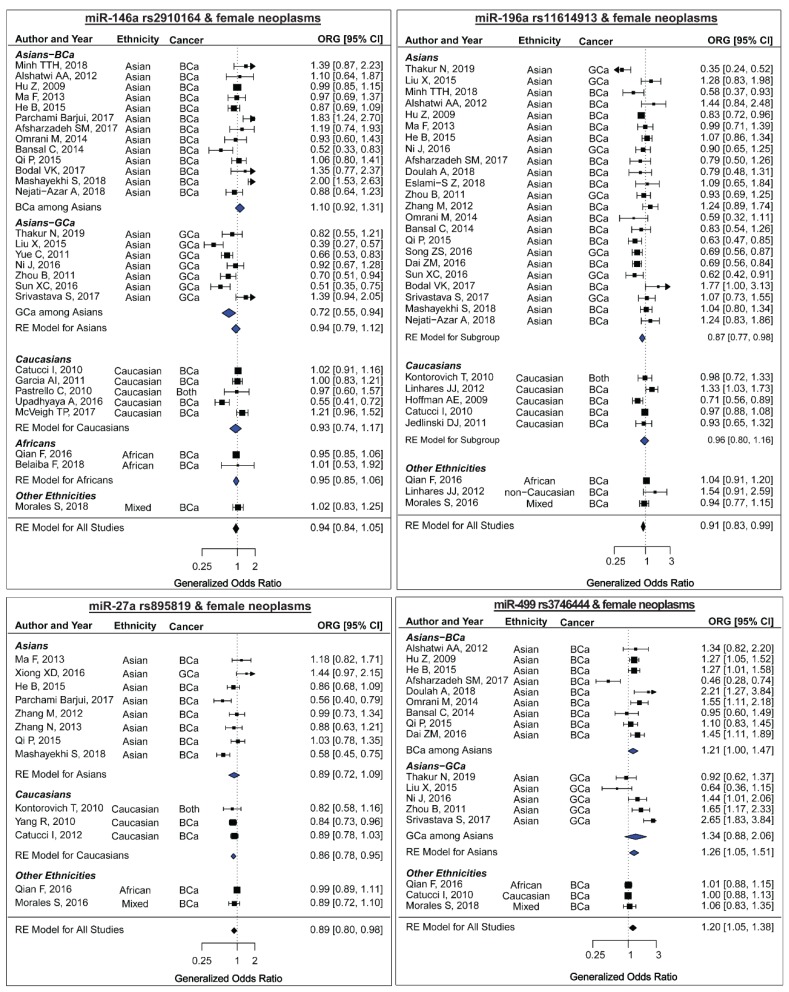
Forest plots of the meta-analysis between miR-146a rs2910164 (top-left), miR-196a2 rs11614913 (top-right), miR-27a rs895819 (bottom-left), and miR-499 rs3746444 (bottom-right) and risk of female neoplasms. The x-axes represent Generalized Odds Ratio (ORG).

**Figure 3:**
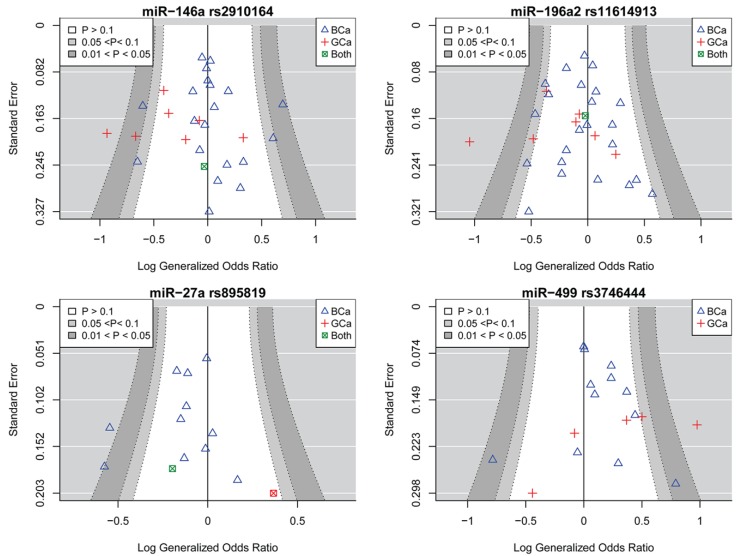
Funnel plots for the meta-analysis between miRNA polymorphisms and female neoplasms. Top-left: miR-146a rs2910164, top-right: miR-196a2 rs11614913, bottom-left: miR-27a rs895819, bottom-right: miR-499 rs3746444. The x-axes represent logarithm of generalized odds ratio and y-axes represent standard error.

Twenty-eight association studies with a total of 11,071 cases and 12,312 controls were included in the meta-analysis of miR-146a rs2910164 and the risk of female neoplasms (Table 1 and Figure 2) [26,28,32,33,34,35,36,38,42,46,47,50,52,55,60,63,67,68,69,70,73,74,77,78,80,81,83,92]. Among these, there were studies that were carried out among Asians (*n*: 20), Caucasians (*n*: 5), Africans (*n*: 1), or South Americans (*n*: 1). The genotype counts among the control group of six studies deviated from Hardy-Weinberg Equilibrium (HWE) expectations [36,46,52,70,78,92]. The meta-analysis by pooling ORGs found no significant association between rs2910164 and female neoplasms (Table 2 and Figure 2: top-left panel, ORG (95%CI): 0.94 (0.84–1.05), *P*: 0.29). This result was also confirmed by the meta-analysis assuming the genetic models (Table 2). Significant heterogeneity was present in all genetic models, with the most heterogeneous model being the homozygote contrast (Table 2). Meta-regression showed that type of cancers (i.e., BCa or GCa) significantly explains a part of the heterogeneity observed in the ORG model (R^2^: 22.27%, *P-value* for the test of moderators: 0.01). No statistical or visual evidence for the asymmetry of funnel plots was observed (All *P-values* > 0.05, Table 2, Figure 3: top-left panel). Subgroup analyses (Table 3) showed that miR-146a rs2910164 might not be associated with female cancers among Asians, Caucasians, or Africans. Moreover, this polymorphism was associated with the risk of BCa neither in Asians nor in Caucasians (Table 3). In the GCa subgroup, pooling ORGs of studies yielded a significant association (Table 3, ORG (95%CI): 0.71 (0.54–0.93)). This indicates that, with the C allele being the mutant and the G being the reference, the mutational load of miR-146a rs2910164 is associated with a lower risk of GCa. Concordantly, the recessive model also yielded a significant association in the GCa subgroup (Table 3), which suggests that women carrying the CC genotype of the studied polymorphism had a lower risk of gynecological cancers than those carrying at-least one G allele. The subgroup analysis that was based on the quality of studies showed no difference among the high-quality or the low-quality studies, which confirms that the results of the overall analysis might not be influenced by low-quality studies. Moreover, the potential influence of excluding HWE-deviated studies was evaluated and the results showed that excluding such studies did not alter the main conclusions of the meta-analysis (ORG (95%CI): all studies 0.94 (0.84–1.05), HWE studies 0.90 (0.79–1.02)). The ORG method has shown superior power in detecting the association when there is a deviation from HWE [94]. Using the approach that was described in the methods section, the overall ORG model was screened and the study by Mashayekhi [32] was identified as a potential influential study. The exclusion of this study led to a reduction of the heterogeneity (tau^2^ from 0.064 to 0.045). However, the conclusion of the overall ORG model did not alter (ORG (95%CI) after exclusion: 0.91 (0.82–1.01), *P*: 0.08, before exclusion: 0.94 (0.84–1.05), *P*: 0.29). Moreover, this study [32] was also identified as a contributor to the heterogeneity of the BCa subgroup and its exclusion led to a significant reduction in heterogeneity (tau^2^ from 0.041 to 0.21), with no effect on the conclusion (ORG (95%CI) after exclusion: 0.99 (0.89–1.08), *P*: 0. 82, I^2^: 59.3; before exclusion: 1.03 (0.92-1.15), *P*: 0.58, I^2^: 72.6). 

Rs2910164 substitutes a C nucleotide in the 3′ arm of hsa-miR-146a precursor (MI0000477) with a G nucleotide [97]. This substitution induces a mispairing in the precursor structure and it affects the third base of the seed region of hsa-miR-146a-3p (MIMAT0004608). Accumulating evidence suggests that rs2910164 might influence the maturation and expression of miR-146a in a context dependent paradigm, in which the true effect of either allele might depend on the cell type and the disease status [97]. Observations that support this paradigm include the discrepancies between pathological and normal samples in terms of miR-146a genotype-expression correlation [98,99] and opposite effects of rs2910164 alleles on miR-146a expression in different cell types or diseases [97,98,99,100,101]. Moreover, miRNA-mediated regulation of gene expression has been shown to be highly influenced by cell-type specific conditions [102]. Therefore, further functional studies on specific disease/cell-type should be carried out to further elucidate the rs2910164-induced changes and their effects on disease susceptibility and progress. Apart from the disease susceptibility, miR-146a and rs2910164 have also been shown to influence proliferation, disease progression, and the survival of cancer patients. Tissue overexpression of miR-146a-5p has been shown to contribute to the proliferation in BCa patients [103]. Moreover, in basal-like BCa cells, the overexpression of miR-146a confers enhanced tumorigenic potential in association with altered p53 status [104]. Triple negative BCa patients with higher expression of miR-146a have a lower survival rate and poorer prognosis [105]. As the expression of miRNAs is under tight regulation, different candidate mechanisms may explain the upregulation of miR-146a in BCa patients. It is not yet clear to what extent rs2910164 might contribute to the disturbed regulation of miR-146a. Apart from its effect on miR-146a expression, rs2010164 may potentially influence the repertoire of target genes of miR-146a-3p [106]. However, most studies have focused on the leading mature miRNA originated from the 5′ arm of the corresponding hairpin and the importance of the 3′ arm miRNAs is just beginning to emerge [107]. Future experiments may shed more lights on the possible role of miR-146a-3p or miR-146a 5p/3p ratio in the development of female neoplasms and the influence of rs2910164 on miR-146a function. 

### 2.3. miR-196a2 rs11614913 and the Risk of Female Neoplasms

Overall, 31 association studies, including 24 studies on BCa, six studies on GCa, and one study on both BCa and GCa, were included [29,31,32,33,35,38,43,44,46,47,50,52,55,57,60,62,63,65,67,68,70,73,77,78,80,81,82,85,87,92]. The article by Linhares et al. [57] is composed of two studies on separate ethnicities. Among these, there were studies that were carried out among Asians (*n*: 23), Caucasians (*n*: 5), Africans (*n*: 1), South Americans (*n*: 1), or non-Caucasian Brazilians (*n*: 1). The genotype counts among the control group of nine studies deviated from HWE expectations [31,33,50,55,57,60,70,78,92]. A meta-analysis of the association between miR-196a2 rs11614913 and the risk of female neoplasms was carried out while using thirty-one studies, including 11034 cases and 12955 controls, and the results revealed significant associations (Table 2 and Figure 2: top-right panel, pooled ORG (95%CI): 0.91 (0.83–0.99), *P*: 0.03). The results were also significant while assuming the homozygote and the recessive models (Table 2), indicating that women carrying the TT genotype had a lower risk of female neoplasms than those carrying at-least one C allele. No statistical or visual evidence for the asymmetry of funnel plots was observed (All *P-values* > 0.05, Table 2, Figure 3: top-right panel). When subgrouped by ethnicity, the model-free and the genetic model approach both concordantly indicated a significant association between miR-196a2 rs11614913 and the risk of female neoplasms among Asians, but not among Caucasians (Table 4). When subgrouped based on the cancer type, the only significant association was identified in the GCa subgroup, as shown by the summary ORG (ORG (95%CI): 0.78 (0.61–0.99)). This polymorphism was not associated with BCa either among Asians or among Caucasians (Table 4).

**Figure 4:**
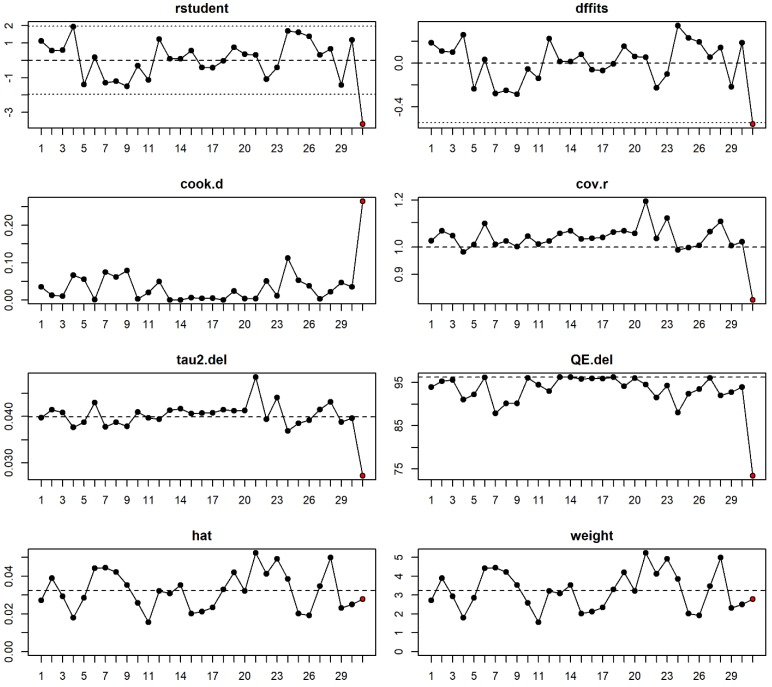
Influential diagnostics for miR-196a2 rs11614913. Each number in the x axes refers to one of the 31 studies included in the meta-analysis. For the 31 studies evaluating the association of miR-196a2 rs11614913 and the risk of female neoplasms, following plots are shown (Please refer to [108,109] for details about each measure): plot of the externally standardized residuals (rstudent), the DFFITS statistic (which is a scaled measure of the change in the predicted value for the ith observation and is calculated by deleting the ith observation), Cook′s distances (which is an estimate of the influence of a data point), covariance ratios, estimates of τ^2^ and test statistics for (residual) heterogeneity when each study is removed in turn, hat values, and weights. A red point indicates the influential study.

Excluding the low-quality studies led to a reduction of the between-study heterogeneity in all contrasts (Table 4). However, the results of the overall analyses were still significant in both the model-free ORG analysis and the genetic contrasts (Table 4), which indicates that the inclusion of the low-quality studies had no dramatic effect on the conclusion of the overall meta-analysis. Moreover, excluding the HWE-deviated studies had no dramatic effect on the conclusion [all studies pooled ORG (95%CI): 0.90 (0.82–0.99), HWE studies pooled ORG (95%CI): 0.89 (0.82–0.98)]. Influential analysis (Figure 4) indicated that the study 31 (i.e., the study by Thakur [92]) might introduce some additional residual heterogeneity into the ORG model, as removing this study would yield considerably smaller estimates of τ^2^. Moreover, as shown in the plot of Cook′s distances (Figure 4), this study has a considerable influence on the fit of the model. The results of the ORG model and the homozygote model were not stable after excluding the study number 31 (ORG (95%CI) before exclusion: 0.91 (0.83–0.99), *P*: 0.03; after exclusion: 0.92 (0.85–1.01), *P*: 0.08). However, the recessive model was not affected by the exclusion of the mentioned study and the result was still significant while assuming this model (OR (95%CI) before exclusion 0.85 (0.73–0.98), *P*: 0.03, I^2^: 60.8, tau^2^: 0.062; after exclusion: 0.87 (0.78–0.98), *P*: 0.03, I^2^: 47.6, tau^2^: 0.037). 

The precursor miRNA originated from hsa-mir-196a-2 locus generates two mature miRNAs, miR-196a-5p and miR-196a-3p. The studied polymorphism, which resided in miR-196a-3p, can affect the expression and targeting ability of miR-196a. There is a 4.6 kcal/mol difference in the minimum free energy of the thermodynamic predicted structure of pre-miR-196a-2 with either T or C allele, suggesting that the T allele might reduce the stability of pre-miR-196a-2 [110]. Indeed, experimentations have shown that the T allele may diminish the processing of the pre-miRNA to its mature form and reduce the expression of miR-196a-2 as compared to the C allele [24,111]. In MCF-7 BCa cells, the transfection of pre-mir-196a-C led to higher mature mir-196a-2 expression as compared to cells that were transfected with pre-mir-196a-T vector [82]. Although rs11614913 is resided outside of the seed region of miR-196a-3p, studies have shown that this polymorphism might affect binding of this mature miRNA and alter the repertoire of target genes of miR-196a-2 [82]. Expression microarray analysis of MCF-7 BCa cells that were transfected with either C or T allele suggested that rs11614913 might influence the repertoire of target genes of miR-196a-2 [82]. Similar results were obtained in other cancers [111].

### 2.4. miR-27a rs895819 and the Risk of Female Neoplasms

Thirteen association studies with a total of 6743 cases and 8461 controls were included in the meta-analysis of miR-27a rs895819 and the risk of female neoplasms (Table 1) [32,36,38,46,47,51,53,55,58,65,66,85,87]. Among the included studies, there were studies that were carried out among Asians (*n*: 8), Caucasians (*n*: 3), Africans (*n*: 1), or South Americans (*n*: 1). Most of the studies evaluated the risk of BCa and only one study assessed GCa. In one study, the genotype distribution of the control group deviated from HWE [85]. A meta-analysis by pooling ORGs revealed as a significant association between miR-27a rs895819 and female neoplasms [Table 2 and Figure 2: bottom-left panel, ORG (95%CI): 0.89 (0.80–0.98), *P*: 0.02]. However, the results of pooling effect sizes under genetic models showed no significant association (Table 2). There were significant heterogeneities in all analyzed genetic models. No statistical or visual evidence for the asymmetry of funnel plots was observed (All *P-values* > 0.05, Table 2, Figure 3: bottom-left panel). When subgrouped by ethnicity, significant associations between miR-27a rs895819 and female neoplasms were observed among Caucasians, but not among Asians (Table 5). This finding suggests the protective role of the rs895819-G allele as compared to the A allele in Caucasians and it indicates that Caucasian women carrying at-least one G allele have a lower risk of female neoplasms than those carrying the AA genotype (Table 5). MiR-27a rs895819 was also associated with the risk of BCa in both the model-free analysis and in the genetic contrasts (Table 5). When BCa studies were subgrouped according to the ethnicity, a significant association between miR-27a rs895819 and the BCa risk was only observed among Caucasians but not among Asians (Table 5). This might indicate the ethnic-dependent effect of miR-27a rs895819 on the BCa risk. However, it should be noted that the Caucasian studies recruited larger sample sizes relative to the Asian studies (Table 5, three Caucasian studies with 2401/3197 samples relative to seven Asian studies with 2062/2012 samples). Therefore, association studies recruiting larger samples are needed to confirm the findings in Asians. Excluding the low-quality studies did not alter the results of genetic contrasts (Table 5). However, the pooled ORG was borderline non-significant after excluding low-quality studies [pooled ORG (95%CI) all studies: 0.88 (0.79–0.98), high quality studies 0.90 (0.81–1.00)]. Excluding the HWE-deviated studies did not influence the significance of the summary ORG [pooled ORG (95%CI): all studies 0.88 (0.79–0.98), HWE studies 0.88 (0.78–0.99)]. No individual study was identified as being influential. 

### 2.5. miR-499 rs3746444 and the Risk of Female Neoplasms

Eighteen studies containing 7627 cases and 9489 controls were included in the meta-analysis of the association between miR-499 rs3746444 and the risk of female neoplasms [28,31,35,37,38,44,46,47,50,52,60,63,67,68,78,80,81,92]. As the genotype distributions were not reported in the manuscript, the study by Kabirizadeh et al. was only included in the allele contrast [37]. Among the included studies, there were studies that were carried out among Asians (*n*: 15), Caucasians (*n*: 3), Africans (*n*: 1), or South Americans (*n*: 1). Moreover, most studies (*n*: 14) evaluated the risk of BCa and only a few studies (*n*: 4) focused on GCa. In four studies, the genotype distributions of the control group significantly deviated from the HWE expectations [50,52,63,92]. For the study by Kabirizadeh et al. the computation of HWE test statistics were not possible due to insufficient reported data [37]. The summary ORG was significant [ORG (95%CI): 1.20 (1.05–1.38), *P* < 0.01], which indicates that, with the C allele being the mutant and the T allele being the reference, the mutational load of miR-499 rs3746444 is implicated in increased susceptibility to female neoplasms. The pooling of effect sizes under genetic models revealed that rs3746444 was associated with the risk of female neoplasms while assuming the homozygote (CC vs. TT) and the allelic models (C vs. T) (Table 2), suggesting that the rs3746444-C allele increases the risk of female neoplasms as compared to the T allele. No statistical or visual evidence for the asymmetry of funnel plots was observed (All *P-values* > 0.05, Table 2 and Figure 3: bottom-right panel). Moreover, miR-499 rs3746444 was found to be associated with female neoplasms among Asians [Table 6, ORG (95%CI): 1.26 (1.05–1.51), C vs. T OR (95%CI): 1.3 (1.05–1.61)]. According to the summary ORGs, miR-499 rs3746444 was associated with a slightly increased risk of BCa [1.14 (1.00–1.30)], but not with the risk of GCa [1.34 (0.87–2.05)]. The association between rs3746444 and the BCa risk did not remain significant after excluding the few non-Asian studies [BCa among Asians ORG (95%CI): 1.21 (0.99–1.47), Table 6]. The exclusion of non-Asian studies led to a significant reduction in the total number of cases and controls (Table 6). The studies by Qian [38] and Catucci [67] are the two large-scale studies carried out among non-Asians (Africans and Caucasians, respectively). This might indicate that the subtle effect that is imposed by rs3746444 on the risk of BCa is difficult to identify when the sample size decreases. Therefore, sufficient data are not available to judge the possible ethnic-specific effects of rs3746444 on the risk of BCa. More large-scale association studies are needed for elucidating the influence of miR-499 rs3746444 on the risk of BCa across different ethnicities. 

In the sensitivity analysis, excluding the low-quality studies led to a significant diminution of the heterogeneity in all modes of inheritance, especially the homozygote and the recessive models (Table 6). Excluding the low-quality studies did not dramatically influence the summary ORG [ORG (95%CI): all studies 1.20 (1.04–1.38), high-quality studies 1.20 (1.07–1.35)]. Moreover, in the subset of high-quality studies, miR-499 rs3746444 was associated with female neoplasms under the dominant and the recessive models in addition to the homozygote and the allele contrasts (Table 6). These results suggest that the summary ORG was more robust in terms of the sensitivity to the inclusion of the low-quality studies when compared to the genetic models. Excluding the HWE-deviated studies did not influence the magnitude or the significance of the summary ORG [pooled ORG (95%CI): all studies 1.20 (1.04–1.38), HWE studies 1.19 (1.01–1.39)]. No individual study was identified to be influential.

Members of human miR-499 precursor family, hsa-pre-mir-499a and hsa-mir-499b, are encoded from opposite strands of an intronic region of *MYH7B* gene [97,112]. According to the miRBase database [113], four mature miRNAs (namely miR-499b-5p, miR-499b-3p, miR-499a-5p, and miR-499a-3p) are generated from this region; two from each precursor. Recent studies have provided evidence supporting the functional role of mature miRNAs originating from both arms of precursor miRNA [114]. While most studies focused on the function of miR-499a-5p, evidence supporting the importance of miR-499a-3p are beginning to emerge [115]. Given that rs3746444 overlaps the seed region of miR-499a-3p on the forward strand and the 3′ portion of miR-499b-5p on the reverse strand, it might influence the processing and/or the 5p/3p balance of both precursors. It has been shown that the rs3746444 C allele might lead to the lower miR-499a-5p expression in breast tissues [60], which suggests a mechanism for the increased BCa risk associated with this allele. However, it is not yet clear whether rs3746444 interfere with miR-499a-3p targeting and what the implications of these changes for female neoplasms. 

### 2.6. miR-423 rs6505162 and the Risk of Female Neoplasms

**Figure 5:**
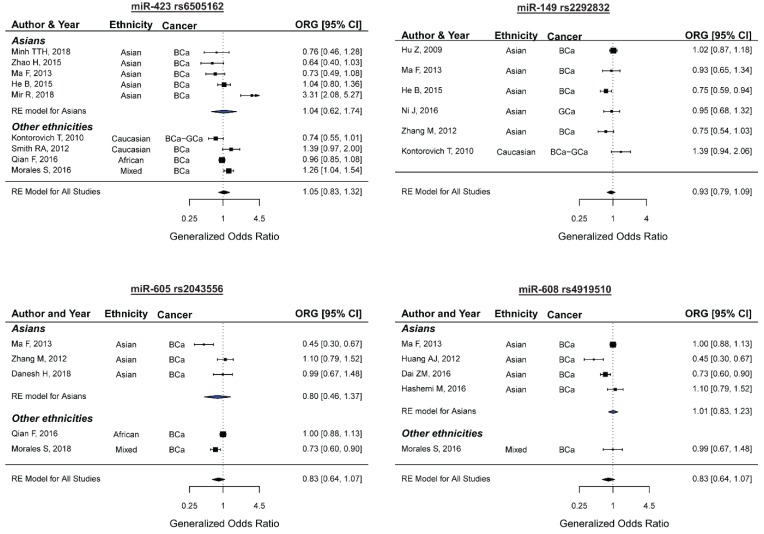
Forest plots for meta-analyses between miR-423 rs6505162 (top-left), miR-149 rs2292832 (top-right), miR-605 rs2043556 (bottom-left), and miR-608 rs4919510 (bottom-right) and risk of female neoplasms.

Nine studies containing 3505 cases and 4273 controls were included in the meta-analysis of the association between miR-423 rs6505162 and the risk of female neoplasms [38,47,55,61,65,72,77,85,88]. Among the included studies, there were studies carried out among Asians (*n*: 5), Caucasians (*n*: 2), Africans (*n*: 1), or South Americans (*n*: 1). Eight studies explored the association of rs6505162 and BCa and one study evaluated both BCa and GCa. In one study, the genotype distributions of the control group significantly deviated from the HWE expectations [72]. Meta-analysis using the summary ORG and the summary OR under different genetic models showed no evidence of an association between miR-423 rs6505162 and female neoplasms (Table 2 and Figure 5: top-left panel). Significant heterogeneity was present in all models, except the recessive model. The subgroup analysis revealed no significant associations in the BCa and Asians subgroups. Moreover, excluding the HWE-deviated study did not dramatically alter the summary ORG (pooled ORG (95%CI): all studies 1.04 (0.83–1.31), HWE studies 0.94 (0.80–1.12)).

### 2.7. miR-149 rs2292832 and the Risk of Female Neoplasms

Six studies containing 2211 cases and 2422 controls were included in the meta-analysis of the association between miR-149 rs2292832 and the risk of female neoplasms [47,55,65,68,80,87]. Among the included studies, there were studies that were carried out among Asians (*n*: 5) or Caucasians (*n*: 1). Five studies explored the association of miR-149 rs2292832 and BCa and one study evaluated both BCa and GCa. In two studies, the genotype distributions of the control group significantly deviated from the HWE expectations [55,65]. Meta-analysis using the summary ORG and the summary OR under different genetic models showed no evidence of an association between miR-149 rs6505162 and female neoplasms (Table 2 and Figure 5: top-right panel). Significant heterogeneity was present in the homozygote (τ: 0.29), the recessive (τ: 0.21), and the allelic (τ: 0.15) models (Table 2, all *P-values* < 0.1). Moreover, excluding the HWE-deviated study did not alter the significance of the summary ORG [pooled ORG (95%CI): all studies 0.92 (0.78–1.09), HWE studies 0.87 (0.72–1.04)].

### 2.8. miR-605 rs2043556 and the Risk of Female Neoplasms

Five studies containing 2706 cases and 3804 controls were included in the meta-analysis [28,30,38,55,87]. Among the included studies, there were studies that were carried out among Asians (*n*: 3), Africans (*n*: 1), or South Americans (*n*: 1). All of the studies explored the association between miR-605 rs2043556 and BCa. In three studies, the genotype distributions of the control group significantly deviated from the HWE expectations [28,30,55]. Meta-analysis using the ORG and the OR under different genetic models showed that miR-605 rs2043556 was not associated with BCa in overall studies (Table 2 and Figure 5: bottom-left panel) or BCa among Asians (Table 7). Although the ORG method has been shown to be less sensitive to HWE-deviation [94], it should be noted that the limited number of studies and a high proportion of HWE-deviated studies might prevent drawing a robust conclusion. In addition, this study did not identify any association study exploring the role of miR-605 rs2043556 in susceptibility to the gynecological cancers. Therefore, more studies are needed to clarify the potential contribution of rs2043556 to female neoplasms.

### 2.9. miR-608 rs4919510 and the Risk of Female Neoplasms

Five studies containing 2115 cases and 3189 controls were included in the meta-analysis [41,44,55,59,85]. Among the included studies, there were studies that were carried out among Asians (*n*: 4) or South Americans (*n*: 1). All of the studies explored the association between miR-608 rs4919510 and BCa. Meta-analysis using the ORG and the OR under the different genetic models showed that miR-608 rs4919510 was not associated with BCa in all studies (Table 2 and Figure 5: bottom-right) or BCa among Asians (Table 7). However, it should be noted that the limited number of studies might prevent drawing a robust conclusion. In addition, this study did not identify any association study exploring the role of rs4919510 in susceptibility to the gynecological cancers. Therefore, more studies are needed to clarify the potential contribution of miR-608 rs4919510 to female neoplasms.

### 2.10. miR-100 rs1834306 and the Risk of Female Neoplasms

**Figure 6:**
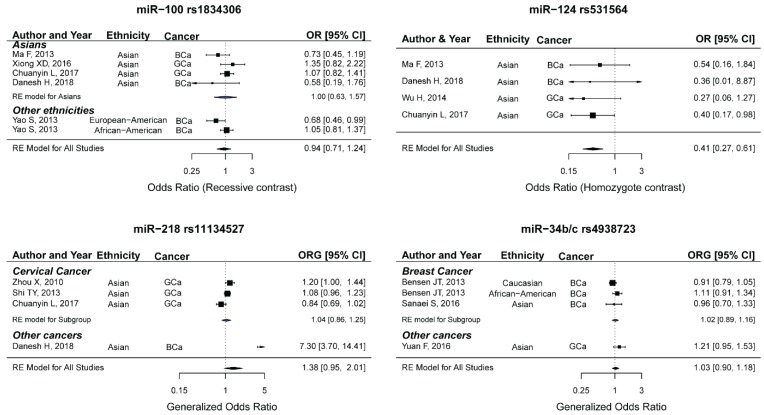
Forest plots for meta-analyses between miR-100 rs1834306 (top-left), miR-124 rs531564 (top-right), miR-218 rs11134527 (bottom-left), and miR-34b/c rs4938723 (bottom-right) and risk of female neoplasms.

Six studies containing 1969 cases and 2192 controls were included in the meta-analysis [30,51,54,55,93]. The article by Yao et al. is composed of two studies on separate ethnicities [54]. Two studies were only included in the recessive model, as sufficient data for calculating the effect size under other models were not provided in the articles. Among the included studies, there were studies that were carried out among Asians (*n*: 4), Africa-Americans (*n*: 1), or European-Americans (*n*: 1). Four studies explored the association of miR-100 rs1834306 and BCa and two studies evaluated GCa. According to the quality scores (Table 1), all of the studies were considered to be high quality. In one studies, the genotype distributions of the control group significantly deviated from the HWE expectations [30]. Meta-analysis using the ORG and the OR under different genetic models showed that miR-100 rs1834306 was not associated with female neoplasms (Table 2 and Figure 6: top-left panel). Moreover, excluding the HWE-deviated study did not alter the significance of the summary ORG [pooled ORG (95%CI): all studies 0.98 (0.84–1.15), HWE studies 0.98 (0.84–1.14)]. It should be noted that more association studies are needed to enable drawing a robust conclusion. 

### 2.11. miR-124 rs531564 and the Risk of Female Neoplasms

Four studies containing 1213 cases and 1312 controls were included in the meta-analysis [30,49,55,93]. All of the studies were performed among Asians. Two studies explored the association between rs531564 and BCa and two studies evaluated GCa (cervical cancer). According to the quality scores (Table 1), three out of four studies were considered to be high quality. The summary ORG revealed no evidence for an association between miR-124 rs531564 and female neoplasms [ORG (95%CI): 0.80 (0.55–1.17)]. However, the summary ORs were significant while assuming the homozygote (Figure 6: top-right panel) and the recessive models (Table 2), which suggests that women with the GG genotype had a lower risk of female neoplasms as compared to those carrying the CC genotype [0.41 (0.27–0.61), *P*: 0.01] or compared to women carrying at least one C allele (Table 2, 0.72 (0.53–0.99), *P*: 0.04). Excluding the low-quality study did not influence the conclusion and the results remained significant under the homozygote and the recessive models (Table 7). Future experiments may benefit from performing genetic association studies among non-Asian ethnicities and exploring the contribution of rs531564 to the risk of other types of female neoplasms. More studies are needed to draw definite conclusions regarding the contribution of this polymorphism to the susceptibility to female neoplasms. 

### 2.12. miR-218 rs11134527 and the Risk of Female Neoplasms

Four studies containing 3134 cases and 2966 controls were included in the meta-analysis [30,56,89,93]. All of the studies were carried out among Asians. One study explored the association between miR-218 rs11134527 and BCa and three studies evaluated GCa (cervical cancer). According to the quality scores (Table 1), all of the studies were considered to be high-quality. A meta-analysis using the ORG revealed no evidence for an association between rs11134527 and female neoplasms (Table 2 and Figure 6: bottom-left panel, ORG (95%CI): 1.38 (0.95–2.01)). Consistently, the summary ORs assuming the genetic models were not significant (Table 2), which suggests that this polymorphism does not contribute to the risk of female neoplasms. When the three studies on cervical cancer were pooled, no significant association was observed (Table 7). Given that only one study was focused on BCa, a definite conclusion regarding the association between miR-218 rs11134527 and BCa might not be drawn from this meta-analysis. Moreover, all of the studies were carried out among Asians and there are no data regarding the possible involvement of miR-218 rs11134527 in the susceptibility to female neoplasms among other ethnicities. Although the current data suggest the lack of association between rs11134527 and female neoplasms among especially cervical cancer Asians, performing more genetic association studies is necessary to obtain a more robust conclusion. 

### 2.13. miR-34b/c rs4938723 and the Risk of Female Neoplasms

Four studies containing 2536 cases and 2535 controls were included in the meta-analysis [39,40,91]. The article by Bensen et. al. is composed of two studies on separate ethnicities [91]. There were studies carried out among Asians (*n*: 2), Caucasians (*n*: 1), and African-Americans (*n*: 1). Three studies explored the association between miR-34b/c rs4938723 and BCa and one study evaluated GCa (i.e., cervical cancer). A meta-analysis using the ORG revealed no evidence for an association between miR-34b/c rs4938723 and female neoplasms [Table 2 and Figure 6: bottom-right panel, ORG (95%CI): 1.03 (0.90–1.18)]. Consistently, the summary ORs assuming the genetic models were not significant (Table 2), which suggests that this polymorphism does not contribute to the risk of female neoplasms. Excluding the low-quality study did not influence the overall results (high-quality studies ORG (95%CI): 0.95 (0.80–1.13), I^2^: 60.30). No significant association was observed when the three studies on BCa were pooled (Table 7). Given that only three studies were focused on BCa, a definite conclusion regarding the association between miR-34b/c rs4938723 and BCa might not be drawn from this meta-analysis. Although the current data suggest the lack of association between miR-34b/c rs4938723 and female neoplasms especially cervical cancer, performing more genetic association studies is necessary for obtaining a more robust conclusion. 

### 2.14. miR-26a-1 rs7372209, miR-373 rs12983273, miR-618 rs2682818 and the Risk of Female Neoplasms

**Figure 7:**
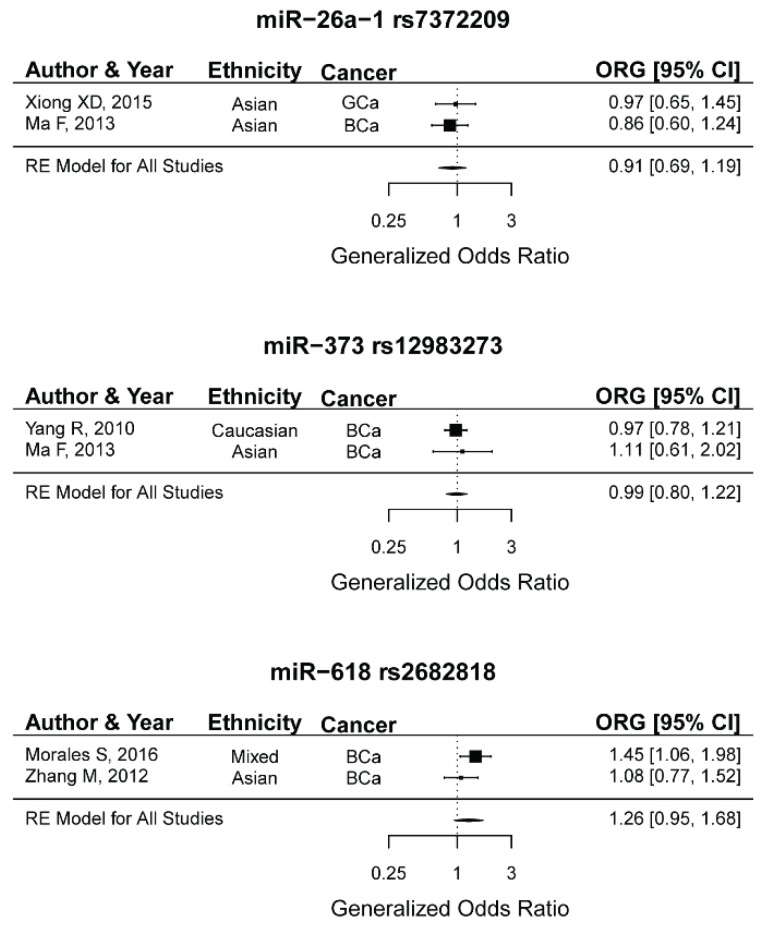
Forest plots for meta-analyses between miR-26a-1 rs7372209 (top), miR-373 rs12983273 (middle), and miR-618 rs2682818 (bottom) and risk of female neoplasms.

Two studies were included in the meta-analysis of the association between each of these miRNA polymorphisms and risk of female neoplasms (Table 2) (rs7372209 [51,55], rs12983273 [55,66], rs2682818 [85,87]). In each case, the included studies were compatible with HWE expectations and considered to be high-quality according to the quality scores (Table 1). The meta-analyses using either the summary ORGs or the summary ORs under the genetic models yielded no significant association between these polymorphisms and female neoplasms (Table 2 and Figure 7). It should be noted that the heterogeneity might not be precisely estimated in the presence of a limited number of studies. Therefore, the results should be treated with caution. Future association studies may shed more lights on the contribution of these miRNA polymorphisms to the risk of female neoplasms.

In conclusion, this study systematically identified 65 miRNA polymorphisms that were evaluated for possible contribution to the risk of female neoplasms (i.e., breast cancer and gynecological cancers). For the majority of studied polymorphisms (*n*: 50), sufficient data for performing a meta-analysis were not available and, therefore, no conclusion regarding the contribution of these polymorphisms to the risk of female neoplasms was drawn in the current study. The following conclusions may be obtained based on the results of this study concerning 15 miRNA polymorphisms that were included in the meta-analysis. For most miRNA polymorphisms, breast cancer was the most studied cancer, followed by the cervical or ovarian cancer, among studies that were included in the meta-analysis. Only a few studies were focused on other types of gynecological cancers and different types of gynecological cancers were less studied than breast cancer. Moreover, it should be noted that this study did not adjust for covariates, like age and gender or interaction with environmental factors, and it was based on unadjusted summary effects of the original studies. Designing a flexible platform for data harmonization might provide the feasibility of performing meta-analysis of individual patient data, which allows for the testing of interactions with covariates [116]. The results of this meta-analysis suggest that miR-146a rs2910164 is implicated in the susceptibility to gynecological cancers. The load of the rs2910164-C allele could be associated with a decreased risk of gynecological cancers. This study also suggests that the miR-196a2 rs11614913-T allele has a moderate protective effect against the development of female neoplasms in Asians but not in Caucasians. This moderate effect vanishes in the breast cancer subgroup, possibly due to the reduction of sample size (i.e., the total number of cases and controls). The load of the miR-196a2 rs11614913-T allele could be associated with a decreased risk of gynecological cancers. Regarding miR-27a rs895819, the G allele might pose a protective effect against female neoplasms, especially against breast cancer among Caucasians. The C allele of miR-499 rs3746444 may slightly increase the risk of female neoplasms especially breast cancer. The G allele of miR-124 rs531564 might be associated with a lower risk of female neoplasms under the homozygote and the recessive models. However, larger samples are needed to confirm this finding. The current evidences do not support the association of the remaining polymorphisms and the risk of female neoplasms (i.e., miR-423 rs6505162, miR-149 rs2292832, miR-605 rs2043556, miR-608 rs4919510, miR-100 rs1834306, miR-218 rs11134527, miR-34b/c rs4938723, miR-26a-1 rs7372209, miR-373 rs12983273, and miR-618 rs2682818).

## 3. Methods

### 3.1. Search Strategy 

Embase, PubMed, ScienceDirect, and Scopus were searched according to specific search tips of each database to identify all potentially eligible publications databases (last search: 9 March 2019). The following keywords or MeSH terms were used. (“Cervical Cancer” OR “Uterine Cervical Neoplasms” OR “Cervix Uteri Cancer” OR “Ovarian Cancer” OR “Fallopian Tube Neoplasms” OR “Endometrial Neoplasms” OR “Uterine Neoplasms” OR “Uterine Serous Carcinoma” OR “Corpus Uteri Cancer” OR “Vaginal Neoplasms” OR “Vulvar Neoplasms” OR “Breast Cancer” OR “Breast Carcinoma” OR “Breast Neoplasms”) AND (miRNA OR microRNA OR pre-mir OR miR) AND (“Single nucleotide polymorphism” OR SNP OR variant OR variation OR polymorphism OR mutation OR locus). References of previous meta-analysis, review articles, or other relevant articles were also manually screened to identify potentially eligible articles. This meta-analysis was carried out in accordance with the Preferred Reporting Items for Systematic Reviews and Meta-Analyses (PRISMA) statement [117].

### 3.2. Inclusion and Exclusion Criteria

Studies meeting all of the following criteria were included: (1) a case-control study evaluating the association between a polymorphism in any miRNA gene and susceptibility to female neoplasms (ICD-10: C50, C51–C58), including breast cancer (ICD-10: C50) and tumors of the female genital tract; (2) availability of the genotype count data for estimating the odds ratio (OR) and its 95% confidence interval (95%CI); (3) the full-text article is written in English; and, (4) a minimum of two studies per each miRNA polymorphism should be met to include the polymorphism in the meta-analysis. Studies that met any of the following criteria were excluded: (1) meta-analysis, review articles or abstracts; (2) duplicate publications; (3) studies on animals or cell-lines; (4) studies without a case-control design; (5) studies that did not report genotype counts or allele frequencies; (6) studies investigating survival, progression, or severity of the disease; and, (7) the article was not written in English.

### 3.3. Data Extraction and Quality Assessment

Each eligible study was screened and the following items were recorded: the first author, publication year, country, ethnicity, the cancer type, the source of controls, the miRNA name, polymorphism ID, genotyping method, genotype counts for each SNP, and number of cases and controls recruited. The quality of each study was assessed while using a modified version of quality assessment criteria for genetic association studies used elsewhere [8,118,119] that scores between 0 (lowest) to 15 (highest). Studies that were scored equal to or less than eight were regarded low quality, while those with scores of nine or higher were regarded high quality.

### 3.4. Data Analysis

Associations of miRNA polymorphisms with female neoplasms are represented with pooling odds ratios (ORs) and their 95% confidence intervals (95% CIs) assuming five genetic models (homozygote BB vs. AA, heterozygote AB vs. AA, dominant BB+AB vs. AA, recessive BB vs. AA+AB, and allelic B vs. A). Moreover, the generalized odds ratio (ORG), which is a model-free measure of genetic risk effect, was also used to evaluate the association between miRNA polymorphisms and female neoplasms [96]. The ORG expresses the association by estimating the overall risk effect while considering the complete genotype distribution and it indicates whether the mutational load of a polymorphism is involved in disease susceptibility [94,96]. The chi-squared based Q test and I^2^ were used to assess the significance of heterogeneity and the Z test was performed to assess the significance of pooled ORs (*P-value* < 0.05 was considered to be statistically significant). Considerable heterogeneity was expected between the studies because of the differences in sources of the control group and other sample characteristics. Therefore, the random-effects model (RE) with the DerSimionian–Laird estimator was used to calculate the summary effects in all cases [120]. In the case of genetic models, the method by Hartung and Knapp was used for adjusting the test statistics and confidence intervals [121].

Univariate meta-regression was carried out to identify potential sources of heterogeneity. Subgroup analyses that were based on the cancer type, ethnicity, and the study quality were also performed. When examining and interpreting the asymmetry of funnel plots, the recommendations of other investigators have been followed [122]. As statistical tests of the funnel plot asymmetry have low test power to distinguish the chance from the real asymmetry when there are fewer than 10 studies in the meta-analysis, such tests were only performed for meta-analysis of ≥10 studies. The method that was proposed by Harbord et al. [123], which is a weighted linear regression test utilizing efficient score and score variance, was used to assess the asymmetry in funnel plots when the estimated heterogeneity variance of log odds ratios, τ^2^, was below 0.1 (i.e., in the absence of substantial heterogeneity). In the presence of substantial heterogeneity (i.e., when τ^2^ > 0.1), the method that was proposed by Rücker et al. [124], an arcsine test that explicitly models between-study heterogeneity, was employed to prevent issues regarding false positive results that may raise in the presence of high heterogeneity. Funnel plots were visually inspected to further assist the interpretation of the mentioned statistical tests. 

Genotype frequencies of polymorphisms in the control group of each study were assessed for the departure from HWE expectations while using the exact goodness of fit test. The following approach was employed to deal with studies in which the genotype counts in the control groups deviated from HWE expectations (i.e., HWE-deviated studies). HWE-deviated studies were not excluded from the meta-analyses. However, a sensitivity analysis was performed to evaluate the possible influence of excluding HWE-deviated studies on the pooled ORG and corresponding 95% CIs. When more than ten studies were included in the meta-analysis, influential study diagnostics for the model-free approach were computed using the metafor package for R [125]. The following diagnostic measures were computed and plotted: externally standardized residuals (rstudent), DFFITS values, Cook′s distances (cook.d), covariance ratios (cov.r), DFBETAS values, the estimates of τ^2^ when each study is removed in turn, the test statistics for (residual) heterogeneity when each study is removed in turn, the diagonal elements of the hat matrix, and the weights (in %) that are given to the observed outcomes during the model fitting [108,125]. The DIFFITS value of a study represents how much the predicted pooled effect changes after excluding this study [109]. Cook′s distance measures the effect of deleting a given observation by calculating the distance between the value once the study is included as compared to when it is excluded [109]. The covariance ration is the determinant of the variance-covariance matrix of the parameter estimates when the study is removed, divided by the determinant of the variance-covariance matrix of the parameter estimates when the full dataset is considered [109]. A study was considered to be probably ‘influential’ if at least one of the following was true [108,125]: (i) The absolute DFFITS value is larger than 3√(p/(k-p)), where p is the number of model coefficients and k is the number of studies. (ii) The lower tail area of a chi-square distribution with p degrees of freedom cut off by the Cook′s distance is larger than 50%. (iii) The hat value is larger than 3(p/k). (iv) Any DFBETAS value is larger than 1. The ORG and its 95% CI were calculated using the ORGGASMA (http://biomath.med.uth.gr) [96]. All other statistical analyses were carried out while using the Meta package for R (R version 3.5.2) [126]. 

## Figures and Tables

**Table 1:** Main characteristics of studies included in the meta-analysis.

miRNA Polymorphism	Author	Cancer	Ethnicity	Genotyping	Source ^a^	Cases ^b^	Controls ^b^	Quality ^c^
miR-100-rs1834306	[93]	CCa	As.	TaqMan	HB	171/299/139	168/289/126	11
miR-100-rs1834306	[30]	BCa	As.	RFLP	HB	52/207/5	46/226/9	9
miR-100-rs1834306	[55]	BCa	As.	MA	BD	60/93/38	55/87/48	11
miR-100-rs1834306	[51]	CCa	As.	LDR	HB	38/38/27	127/203/87	9
miR-100-rs1834306	[54]	BCa	A.A	Illumina	PB	473	412	14
miR-100-rs1834306	[54]	BCa	E.A	Illumina	PB	329	309	14
miR-124-rs531564	[93]	CCa	As.	TaqMan	HB	17/144/448	7/118/458	11
miR-124-rs531564	[30]	BCa	As.	RFLP	HB	227/37/0	245/34/1	10
miR-124-rs531564	[55]	BCa	As.	MA	BD	126/52/4	136/45/8	11
miR-124-rs531564	[49]	CCa	As.	PCR-LDR	HB	134/22/2	184/66/10	8
miR-146a-rs2910164	[78]	BCa	As.	ARMS	NA	33/61/6	57/84/9	4
miR-146a-rs2910164	[60]	BCa	As.	TaqMan	NA	48/50/2	51/46/3	5
miR-146a-rs2910164	[50]	BCa	As.	RFLP	PB	82/35/4	84/72/8	10
miR-146a-rs2910164	[26]	BCa	Af.	SQ	PB	46/29/8	28/17/5	8
miR-146a-rs2910164	[70]	BCa	As.	RFLP	NA	52/46/0	60/39/0	6
miR-146a-rs2910164	[67]	BCa	Ca.	TaqMan/SQ	BD	860/590/109	1186/838/123	12
miR-146a-rs2910164	[83]	BCa	Ca.	TaqMan	NA	676/388/66	352/220/24	9
miR-146a-rs2910164	[47]	BCa	As.	MA	HB	75/242/133	72/225/153	9
miR-146a-rs2910164	[68]	BCa	As.	RFLP	PB	165/515/329	180/551/362	14
miR-146a-rs2910164	[81]	ECa, OCa	As.	RFLP	NA	101/62/53	12/55/33	7
miR-146a-rs2910164	[55]	BCa	As.	MA	BD	35/94/63	34/93/64	11
miR-146a-rs2910164	[32]	BCa	As.	RFLP	HB	130/178/45	190/145/18	10
miR-146a-rs2910164	[34]	BCa	Ca.	TaqMan	BD	324/171/28	445/199/27	9
miR-146a-rs2910164	[77]	BCa	As.	HRM	BD	7/54/39	22/49/41	9
miR-146a-rs2910164	[28]	BCa	SA	TaqMan	PB	236/165/39	561/410/77	14
miR-146a-rs2910164	[33]	BCa	As.	RFLP	HB	74/84/42	64/94/42	7
miR-146a-rs2910164	[80]	OCa	As.	RFLP	HB	24/75/56	50/161/131	9
miR-146a-rs2910164	[52]	BCa	As.	ARMS	PB	183/45/8	155/39/9	9
miR-146a-rs2910164	[36]	BCa	As.	RFLP	NA	153/76/11	178/45/8	5
miR-146a-rs2910164	[69]	BCa, OCa	Ca.	SQ	NA	60/36/5	90/59/6	5
miR-146a-rs2910164	[46]	BCa	As.	TaqMan	HB	146/132/43	126/144/20	6
miR-146a-rs2910164	[38]	BCa	Af.	Illumina	BD	567/802/284	678/972/378	11
miR-146a-rs2910164	[35]	CCa	As.	RFLP	HB	81/85/18	84/72/8	8
miR-146a-rs2910164	[73]	OCa	As.	RFLP	HB	29/62/43	19/103/105	6
miR-146a-rs2910164	[92]	CCa	As.	RFLP	NA	80/49/21	73/49/28	6
miR-146a-rs2910164	[42]	BCa	Ca.	HRM	PB	325/193/28	112/99/35	12
miR-146a-rs2910164	[74]	CCa	As.	RFLP	HB	118/224/105	87/206/150	10
miR-146a-rs2910164	[63]	BCa	As.	RFLP	HB	43/113/70	34/159/116	10
miR-149-rs2292832	[47]	BCa	As.	MA	HB	231/183/36	202/188/60	9
miR-149-rs2292832	[68]	BCa	As.	RFLP	PB	99/460/450	108/503/482	14
miR-149-rs2292832	[65]	BCa, OCa	Ca.	MA	HB	40/40/87	39/30/53	6
miR-149-rs2292832	[55]	BCa	As.	MA	BD	99/69/17	100/60/26	10
miR-149-rs2292832	[80]	OCa	As.	RFLP	HB	26/82/47	55/179/108	9
miR-149-rs2292832	[87]	BCa	As.	RFLP	PB	120/102/23	92/113/24	13
miR-196a2-rs11614913	[78]	BCa	As.	ARMS	NA	34/52/14	38/93/19	4
miR-196a2-rs11614913	[60]	BCa	As.	TaqMan	NA	35/63/2	46/50/4	4
miR-196a2-rs11614913	[50]	BCa	As.	RFLP	PB	68/41/12	85/59/21	9
miR-196a2-rs11614913	[70]	BCa	As.	RFLP	NA	48/47/0	64/35/0	6
miR-196a2-rs11614913	[67]	BCa	Ca.	TaqMan/SQ	BD	766/842/244	1116/1246/377	12
miR-196a2-rs11614913	[44]	BCa	As.	MA	HB	197/265/98	155/284/144	10
miR-196a2-rs11614913	[31]	BCa	As.	ARMS	NA	33/51/14	25/62/13	1
miR-196a2-rs11614913	[29]	BCa	As.	RFLP	HB	53/42/5	56/38/6	7
miR-196a2-rs11614913	[47]	BCa	As.	MA	HB	81/233/136	93/223/134	9
miR-196a2-rs11614913	[82]	BCa	Ca.	MA	HB	181/209/36	166/229/71	11
miR-196a2-rs11614913	[68]	BCa	As.	RFLP	PB	239/483/287	218/517/358	14
miR-196a2-rs11614913	[62]	BCa	Ca.	RFLP	PB	68/86/33	58/82/31	12
miR-196a2-rs11614913	[65]	BCa, OCa	Ca.	MA	HB	106/110/53	78/88/39	7
miR-196a2-rs11614913	[57]	BCa	Ca.	TaqMan	HB	83/148/94	94/144/66	8
miR-196a2-rs11614913	[57]	BCa	Non-Ca.	TaqMan	HB	11/29/23	33/21/30	5
miR-196a2-rs11614913	[81]	ECa, OCa	As.	RFLP	NA	25/133/58	23/49/28	7
miR-196a2-rs11614913	[55]	BCa	As.	MA	BD	44/92/54	49/79/59	10
miR-196a2-rs11614913	[32]	BCa	As.	RFLP	HB	142/169/42	149/158/46	10
miR-196a2-rs11614913	[77]	BCa	As.	HRM	BD	37/49/14	29/55/28	9
miR-196a2-rs11614913	[85]	BCa	SA	TaqMan	PB	192/191/57	342/351/114	15
miR-196a2-rs11614913	[33]	BCa	As.	RFLP	HB	36/128/36	26/160/14	6
miR-196a2-rs11614913	[80]	OCa	As.	RFLP	HB	32/82/41	66/176/100	9
miR-196a2-rs11614913	[52]	BCa	As.	ARMS	PB	218/18/0	178/25/0	10
miR-196a2-rs11614913	[46]	BCa	As.	TaqMan	HB	34/119/168	17/88/185	7
miR-196a2-rs11614913	[38]	BCa	Af.	Illumina	BD	1120/503/34	1395/579/54	11
miR-196a2-rs11614913	[43]	OCa	As.	RFLP	HB	121/247/111	86/203/142	10
miR-196a2-rs11614913	[35]	CCa	As.	RFLP	HB	20/93/71	21/81/62	8
miR-196a2-rs11614913	[73]	OCa	As.	RFLP	HB	29/66/29	34/116/77	6
miR-196a2-rs11614913	[92]	CCa	As.	RFLP	NA	75/58/17	42/51/57	6
miR-196a2-rs11614913	[87]	BCa	As.	RFLP	PB	11/89/148	17/93/133	13
miR-196a2-rs11614913	[63]	BCa	As.	RFLP	HB	46/123/57	58/169/82	10
miR-218-rs11134527	[93]	CCa	As.	TaqMan	HB	233/294/92	242/273/68	11
miR-218-rs11134527	[30]	BCa	As.	RFLP	HB	206/59/0	269/10/0	10
miR-218-rs11134527	[56]	CCa	As.	TaqMan	HB	588/752/225	512/638/241	11
miR-218-rs11134527	[89]	CCa	As.	RFLP	PB	268/316/101	247/339/127	13
miR-26a-1-rs7372209	[55]	BCa	As.	MA	BD	109/64/19	99/74/18	11
miR-26a-1-rs7372209	[51]	CCa	As.	LDR	HB	57/36/10	221/167/29	9
miR-27a-rs895819	[58]	BCa	Ca.	TaqMan	BD	547/388/90	803/633/157	10
miR-27a-rs895819	[47]	BCa	As.	MA	HB	251/165/34	232/181/37	9
miR-27a-rs895819	[65]	BCa, OCa	Ca.	MA	HB	141/112/14	91/82/15	8
miR-27a-rs895819	[55]	BCa	As.	MA	BD	97/76/16	106/70/14	11
miR-27a-rs895819	[32]	BCa	As.	RFLP	HB	167/156/30	127/155/71	10
miR-27a-rs895819	[85]	BCa	SA	TaqMan	PB	245/166/29	432/298/77	14
miR-27a-rs895819	[36]	BCa	As.	RFLP	NA	156/68/16	113/99/19	6
miR-27a-rs895819	[46]	BCa	As.	TaqMan	HB	101/159/61	95/139/56	7
miR-27a-rs895819	[38]	BCa	Af.	Illumina	BD	376/833/448	455/1025/548	11
miR-27a-rs895819	[51]	CCa	As.	LDR	HB	48/40/15	223/170/24	9
miR-27a-rs895819	[66]	BCa	Ca.	TaqMan/SQ	HB	576/486/127	605/660/151	11
miR-27a-rs895819	[87]	BCa	As.	RFLP	PB	60/144/41	75/109/59	13
miR-27a-rs895819	[53]	BCa	As.	TaqMan	HB	152/96/16	137/103/15	10
miR-34b-c-rs4938723	[91]	BCa	A.A	SQ	PB	362/317/63	343/257/58	14
miR-34b-c-rs4938723	[91]	BCa	Ca.	SQ	PB	496/563/144	430/503/155	14
miR-34b-c-rs4938723	[39]	BCa	As.	RFLP	PB	125/115/23	100/106/15	8
miR-34b-c-rs4938723	[40]	CCa	As.	RFLP	HB	117/175/36	242/258/68	10
miR-373-rs12983273	[55]	BCa	As.	MA	BD	161/25/1	160/21/2	11
miR-373-rs12983273	[66]	BCa	Ca.	TaqMan/SQ	HB	566/184/18	540/175/22	11
miR-423-rs6505162	[47]	BCa	As.	MA	HB	292/142/16	299/129/22	9
miR-423-rs6505162	[65]	BCa, OCa	Ca.	MA	HB	97/114/56	55/92/49	8
miR-423-rs6505162	[55]	BCa	As.	MA	BD	127/57/8	110/69/10	11
miR-423-rs6505162	[77]	BCa	As.	HRM	BD	67/34/5	64/49/3	9
miR-423-rs6505162	[72]	BCa	As.	ARMS	NA	25/52/23	81/25/18	5
miR-423-rs6505162	[85]	BCa	SA	TaqMan	PB	125/229/86	284/385/138	15
miR-423-rs6505162	[38]	BCa	Af.	Illumina	BD	90/625/942	119/727/1182	11
miR-423-rs6505162	[61]	BCa	Ca.	HRM	HB	24/95/60	42/80/52	7
miR-423-rs6505162	[88]	BCa	As.	SQ	HB	79/30/5	110/69/10	8
miR-499-rs3746444	[78]	BCa	As.	ARMS	NA	63/33/4	66/65/19	5
miR-499-rs3746444	[60]	BCa	As.	TaqMan	NA	30/62/8	45/40/15	5
miR-499-rs3746444	[50]	BCa	As.	RFLP	PB	80/30/11	106/43/15	9
miR-499-rs3746444	[67]	BCa	Ca.	TaqMan/SQ	BD	950/545/84	1305/742/120	12
miR-499-rs3746444	[44]	BCa	As.	MA	HB	407/135/18	463/109/11	10
miR-499-rs3746444	[31]	BCa	As.	ARMS	NA	35/35/10	63/33/4	2
miR-499-rs3746444	[47]	BCa	As.	MA	HB	184/177/89	203/188/59	9
miR-499-rs3746444	[68]	BCa	As.	RFLP	PB	707/258/44	816/248/29	14
miR-499-rs3746444	[37]	BCa	As.	ASP	HB	43	48	3
miR-499-rs3746444	[81]	ECa, OCa	As.	RFLP	NA	181/35/0	77/23/0	7
miR-499-rs3746444	[28]	BCa	S.A	TaqMan	PB	319/111/10	772/254/22	14
miR-499-rs3746444	[80]	OCa	As.	RFLP	HB	84/53/18	213/110/19	9
miR-499-rs3746444	[52]	BCa	As.	ARMS	PB	131/44/61	130/48/25	9
miR-499-rs3746444	[46]	BCa	As.	TaqMan	HB	152/117/52	141/112/37	7
miR-499-rs3746444	[38]	BCa	Af.	Illumina	BD	1124/463/70	1374/582/72	11
miR-499-rs3746444	[35]	CCa	As.	RFLP	HB	26/78/80	54/76/34	8
miR-499-rs3746444	[92]	CCa	As.	RFLP	NA	25/47/78	21/49/80	6
miR-499-rs3746444	[63]	BCa	As.	RFLP	HB	134/84/8	223/71/15	9
miR-605-rs2043556	[30]	BCa	As.	RFLP	HB	38/211/15	42/221/18	9
miR-605-rs2043556	[55]	BCa	As.	MA	BD	42/51/4	68/81/60	10
miR-605-rs2043556	[28]	BCa	S.A	TaqMan	PB	208/182/50	376/571/101	13
miR-605-rs2043556	[38]	BCa	Af.	Illumina	BD	975/574/108	1186/726/116	11
miR-605-rs2043556	[87]	BCa	As.	RFLP	PB	131/90/27	125/102/11	13
miR-608-rs4919510	[44]	BCa	As.	MA	HB	157/296/107	183/287/113	10
miR-608-rs4919510	[41]	BCa	As.	RFLP	PB	140/20/0	149/43/0	8
miR-608-rs4919510	[59]	BCa	As.	SNPstream	PB	128/381/254	277/684/456	14
miR-608-rs4919510	[55]	BCa	As.	MA	BD	37/98/57	47/82/61	11
miR-608-rs4919510	[85]	BCa	S.A	TaqMan	PB	226/174/40	431/310/66	15
miR-618-rs2682818	[85]	BCa	S.A	TaqMan	PB	359/78/3	699/102/6	15
miR-618-rs2682818	[87]	BCa	As.	RFLP	PB	132/99/13	130/91/11	13

**a:** The source of the control group, either hospital based (HB), blood donor (BD) or population based (PB). **b:** genotypes counts in cases or controls, represented as GG/GC/CC (for miR-146a), CC/CT/TT (miR-196a, miR-26a-1, miR-373), AA/AG/GG (miR-27a, miR-605), TT/TC/CC (miR-499, miR-149, miR-34b/c), CC/CA/AA (miR-423, miR-618), GG/AG/AA (miR-100, miR-218), CC/CG/GG (miR-608, miR-124)) ; **c:** Quality scores. Studies with score ≥ nine were considered high quality. A.A: African American; Af.: African; As.: Asian; BCa: breast cancer; BD: blood donor; Ca.: Caucasian; CCa: cervical cancer; E.A: European American; ECa: endometrial cancer; HB: hospital based; LDR: PCR-LDR (ligase detection reaction); MA: Mass-ARRAY; NA: not available/applicable; OCa: ovarian cancer; PB: population based; SQ: direct sequencing. S.A: South Americans.

**Table 2:** Summary results for meta-analysis of the association between miRNA polymorphisms and the risk of female neoplasms.

Genetic Models	N ^a^	Samples	OR^b^ (95% CI)	*P* ^c^	*P*_Het_^d^	I*^2^*	τ	*P*_bias_^e^
*miR-146a rs2910164*								
Homozygote(CC vs. GG)	28	11071/12312	0.92 (0.7–1.19)	0.5	<0.01	76.8 (66.8–83.8)	0.47	0.66
Heterozygote(GC vs. GG)	28	11071/12312	0.94 (0.79–1.12)	0.49	<0.01	72.1 (59.4–80.9)	0.27	0.54
Dominant(CC+GC vs. GG)	28	11071/12312	0.93 (0.77–1.12)	0.43	<0.01	77.6 (68.1–84.3)	0.3	0.47
Recessive(CC vs. GC+GG)	28	11071/12312	0.96 (0.81–1.14)	0.63	<0.01	63.2 (44.8–75.2)	0.28	0.66
Allelic(C vs. G)	28	11071/12312	0.95 (0.84–1.08)	0.45	<0.01	79.5 (71–85.5)	0.22	0.73
ORG ^f^	28	11071/12312	0.94 (0.84–1.05)	0.29	<0.01	78.34	0.25	-
*miR-196a2 rs11614913*								
Homozygote(TT vs. CC)	31	11034/12955	0.82 (0.68–0.99)	0.04	<0.01	65.2 (49.1–76.2)	0.34	0.90
Heterozygote(CT vs. CC)	31	11034/12955	0.97 (0.86–1.09)	0.55	<0.01	48.3 (21.5–66)	0.17	0.90
Dominant(CT+TT vs. CC)	31	11034/12955	0.92 (0.8–1.06)	0.23	<0.01	62.6 (45–74.6)	0.22	0.87
Recessive(TT vs. CC+CT)	31	11034/12955	0.85 (0.73–0.98)	0.03	<0.01	60.8 (42.0 –73.5)	0.25	0.91
Allelic(T vs. C)	31	11034/12955	0.94 (0.87–1.01)	0.11	<0.01	59.2 (39.4–72.5)	0.14	0.74
ORG	31	11034/12955	0.91 (0.83–0.99)	0.03	<0.01	68.83	0.19	-
*miR-27a rs895819*								
Homozygote(GG vs. AA)	13	6743/8461	0.85 (0.65–1.11)	0.2	<0.05	64.2 (35.3–80.2)	0.28	0.83
Heterozygote(AG vs. AA)	13	6743/8461	0.91 (0.79–1.05)	0.18	<0.05	54.2 (14.3–75.5)	0.15	0.54
Dominant(AG+GG vs. AA)	13	6743/8461	0.89 (0.77–1.03)	0.11	<0.05	57.9 (22–77.2)	0.15	0.67
Recessive(GG vs. AA+AG)	13	6743/8461	0.87 (0.68–1.11)	0.24	<0.05	64.5 (35.8–80.4)	0.26	0.64
Allelic(G vs. A)	13	6743/8461	0.9 (0.8–1.02)	0.09	<0.05	65.7 (38.3–80.9)	0.13	0.73
ORG	13	6743/8461	0.89 (0.80–0.98)	0.02	<0.01	62.2	0.14	-
*miR-499 rs3746444*								
Homozygote(CC vs. TT)	17	7584/9441	1.40 (1.01–1.96)	0.046	<0.01	69.1 (49.1–81.2)	0.43	0.62
Heterozygote(TC vs. TT)	17	7584/9441	1.13 (0.95–1.35)	0.16	<0.01	63.1 (37.8–78.1)	0.2	0.37
Dominant(TC+CC vs. TT)	17	7584/9441	1.2 (0.98–1.46)	0.07	<0.01	72 (54.4–82.8)	0.23	0.24
Recessive(CC vs. TT+TC)	17	7584/9441	1.33 (0.99–1.77)	0.05	<0.01	65.3 (41.9–79.2)	0.37	0.74
Allelic(C vs. T)	18	7627/9489	1.23 (1.03–1.46)	0.02	<0.01	77.9 (65.6–85.9)	0.23	0.23
ORG	17	7584/9441	1.20 (1.05–1.38)	<0.01	<0.01	76.42	0.23	-
*miR-423 rs6505162*								
Homozygote(AA vs. CC)	9	3505/4273	1.18 (0.74–1.88)	0.43	<0.01	67.6 (34.8–83.9)	0.41	-
Heterozygote(AC vs. CC)	9	3505/4273	1.15 (0.67–2)	0.57	<0.01	84.9 (73.1–91.5)	0.48	-
Dominant(AC+AA vs. CC)	9	3505/4273	1.14 (0.68–1.91)	0.58	<0.01	85.5 (74.4–91.8)	0.47	-
Recessive(AA vs. CC+AC)	9	3505/4273	0.98 (0.86–1.12)	0.78	0.43	1 (0–65.2)	0.02	-
Allelic(A vs. C)	9	3505/4273	1.05 (0.77–1.44)	0.72	<0.01	82.4 (68–90.4)	0.26	-
ORG	9	3505/4273	1.05 (0.83–1.32)	0.69	<0.01	82.15	0.30	-
*miR-149 rs2292832*								
Homozygote(CC vs. TT)	6	2211/2422	0.86 (0.57–1.29)	0.39	0.05	55.9 (0–82.3)	0.29	-
Heterozygote(CT vs. TT)	6	2211/2422	0.92 (0.75–1.13)	0.35	0.43	0 (0–74.2)	0	-
Dominant(CT+CC vs. TT)	6	2211/2422	0.92 (0.71–1.18)	0.42	0.17	35.2 (0–74.1)	0.14	-
Recessive(CC vs. TT+CT)	6	2211/2422	0.9 (0.64–1.26)	0.45	0.06	52.7 (0–81.1)	0.21	-
Allelic(C vs. T)	6	2211/2422	0.94 (0.76–1.16)	0.48	0.03	61 (4.5–84)	0.15	-
ORG	6	2211/2422	0.92 (0.78–1.09)	0.36	0.06	52.34	0.14	-
*miR-605 rs2043556*								
Homozygote(GG vs. AA)	5	2706/3804	0.85 (0.24–2.97)	0.74	<0.01	82.3 (59.4–92.3)	0.57	-
Heterozygote(GA vs. AA)	5	2706/3804	0.85 (0.62–1.16)	0.22	<0.01	72.5 (30.9–89)	0.24	-
Dominant(GA+GG vs. AA)	5	2706/3804	0.84 (0.61–1.15)	0.19	<0.01	72.1 (29.9–88.9)	0.23	-
Recessive(GG vs. AA+GA)	5	2706/3804	0.91 (0.25–3.35)	0.85	<0.01	84.3 (64.8–93)	0.58	-
Allelic(G vs. A)	5	2706/3804	0.88 (0.59–1.3)	0.41	<0.01	81.3 (56.5–92)	0.21	-
ORG	5	2706/3804	0.83 (0.64–1.07)	0.15	<0.01	79.97	0.25	-
*miR-608 rs4919510*								
Homozygote(GG vs. CC)	5	2115/3189	1.16 (0.97–1.39)	0.08	0.98	0 (0–0)	0	-
Heterozygote(GC vs. CC)	5	2115/3189	1.09 (0.72–1.65)	0.59	0.05	58.8 (0–84.6)	0.19	-
Dominant(GG+GC vs. CC)	5	2115/3189	1.09 (0.75–1.58)	0.57	0.06	55.2 (0–83.5)	0.17	-
Recessive(GG vs. GC+CC)	5	2115/3189	1.02 (0.89–1.17)	0.71	0.94	0 (0–0)	0	-
Allelic(G vs. C)	5	2115/3189	1.04 (0.86–1.26)	0.57	0.18	35.7 (0–75.9)	0.08	-
ORG	5	2115/3189	1.04 (0.90–1.19)	0.57	0.14	40.73	0.09	-
*miR-100 rs1834306*								
Homozygote(AA vs. GG)	4	1167/1471	0.96 (0.65–1.42)	0.77	0.42	0 (0–83.9)	0	-
Heterozygote(AG vs. GG)	4	1167/1471	0.9 (0.65–1.24)	0.37	0.37	5.1 (0–85.5)	0.05	-
Dominant(AA+AG vs. GG)	4	1167/1471	0.92 (0.72–1.17)	0.35	0.55	0 (0–78)	0	-
Recessive(AA vs. AG+GG)	6	1969/2192	0.94 (0.71–1.24)	0.59	0.15	38.6 (0–75.6)	0.16	-
Allelic(A vs. G)	4	1167/1471	0.97 (0.84–1.12)	0.55	0.62	0 (0–73.9)	0	-
ORG	4	1167/1471	0.98 (0.84–1.15)	0.84	0.41	0	0	-
*miR-124 rs531564*								
Homozygote(GG vs. CC)	4	1213/1312	0.41 (0.27–0.61)	0.01	0.93	0 (0–1.2)	0	-
Heterozygote(GC vs. CC)	4	1213/1312	0.8 (0.34–1.88)	0.47	0.01	71.9 (20.4–90.1)	0.46	-
Dominant(GG+GC vs. CC)	4	1213/1312	0.74 (0.3–1.8)	0.35	<0.01	74.8 (30.1–90.9)	0.48	-
Recessive(GG vs. GC+CC)	4	1213/1312	0.72 (0.53–0.99)	0.04	0.63	0 (0–73.5)	0	-
Allelic(G vs. C)	4	1213/1312	0.79 (0.43–1.44)	0.3	0.03	67.9 (6.5–88.9)	0.28	-
ORG	4	1213/1312	0.80 (0.55–1.17)	0.25	0.01	70.02	0.31	-
*miR-218 rs11134527 G>A*								
Homozygote(AA vs. GG)	4	3134/2966	1.08 (0.47–2.5)	0.73	0.02	75.8 (20.3–92.6)	0.26	-
Heterozygote(AG vs. GG)	4	3134/2966	1.58 (0.35–7.02)	0.4	<0.01	90.9 (79.8–95.9)	0.53	-
Dominant(AA+AG vs. GG)	4	3134/2966	1.57 (0.35–7.03)	0.41	<0.01	91.5 (81.3–96.1)	0.52	-
Recessive(AA vs. AG+GG)	4	3134/2966	1.03 (0.68–1.56)	0.82	0.09	58.2 (0–88.1)	0.12	-
Allelic(A vs. G)	4	3134/2966	1.31 (0.4–4.24)	0.52	<0.01	91.8 (82.3–96.2)	0.29	-
ORG	4	3134/2966	1.38 (0.95–2.01)	0.08	<0.01	92.09	0.34	-
*miR-34b/c rs4938723*								
Homozygote(CC vs. TT)	4	2536/2535	0.93 (0.7–1.23)	0.45	0.47	0 (0–81.7)	0	-
Heterozygote(CT vs. TT)	4	2536/2535	1.09 (0.8–1.48)	0.44	0.1	52.3 (0–84.2)	0.13	-
Dominant(CT+CC vs. TT)	4	2536/2535	1.06 (0.81–1.4)	0.52	0.11	50.5 (0–83.6)	0.12	-
Recessive(CC vs. TT+CT)	4	2536/2535	0.89 (0.71–1.12)	0.21	0.59	0 (0–75.9)	0	-
Allelic(C vs. T)	4	2536/2535	1.01 (0.86–1.19)	0.83	0.23	30.7 (0–74.9)	0.06	-
ORG	4	2536/2535	1.02 (0.89–1.18)	0.67	0.16	41.53	0.08	-
*miR-26a-1 rs7372209*								
Homozygote(TT vs. CC)	2	295/608	1.11 (0.14–9.1)	0.63	0.53	0	0	-
Heterozygote(CT vs. CC)	2	295/608	0.81 (0.55–1.2)	0.09	0.85	0	0	-
Dominant(CT+TT vs. CC)	2	295/608	0.86 (0.44–1.67)	0.21	0.73	0	0	-
Recessive(TT vs. CC+CT)	2	295/608	1.21 (0.17–8.58)	0.43	0.55	0	0	-
Allelic(T vs. C)	2	295/608	0.95 (0.44–2.05)	0.53	0.61	0	0	-
ORG	2	295/608	0.90 (0.69–1.18)	0.48	0.66	0	0	-
*miR-373 rs12983273*								
Homozygote(TT vs. CC)	2	955/920	0.76 (0.18–3.11)	0.24	0.72	0	0	-
Heterozygote(CT vs. CC)	2	955/920	1.02 (0.51–2.07)	0.74	0.63	0	0	-
Dominant(CT+TT vs. CC)	2	955/920	0.99 (0.56–1.78)	0.94	0.67	0	0	-
Recessive(TT vs. CC+CT)	2	955/920	0.76 (0.17–3.29)	0.25	0.71	0	0	-
Allelic(T vs. C)	2	955/920	0.97 (0.64–1.47)	0.51	0.74	0	0	-
ORG	2	955/920	0.98 (0.80–1.21)	0.9	0.67	0	0	-
*miR-618 rs2682818*								
Homozygote(AA vs. CC)	2	684/1039	1.11 (0.41–3.03)	0.41	0.83	0	0	-
Heterozygote(CA vs. CC)	2	684/1039	1.28(0.16–10.28)	0.37	0.19	41.7	0.15	-
Dominant(CA+AA vs. CC)	2	684/1039	1.27 (0.19–8.52)	0.35	0.22	33.7	0.12	-
Recessive(AA vs. CC+CA)	2	684/1039	1.07 (0.33–3.45)	0.59	0.8	0	0	-
Allelic(A vs. C)	2	684/1039	1.22 (0.23–6.39)	0.37	0.22	33.8	0.11	-
ORG	2	684/1039	1.26 (0.94–1.68)	0.11	0.21	35.50	0.12	

**a**: number of studies; **b:** Pooled OR and 95% CI (Random-effect model); **c:** *Pvalue* of the Z-test; **d**: *P-value* of the Q-test; **e**: *Pvalue* of the Harbord test (when τ^2^ < 0.1) or the arcsine test (when τ^2^ > 0.1) for funnel plot asymmetry test. **f**: ORG stands for the generalized odds ratio. For more details, refer to Section 3.4 or ref. [94,95,96].

**Table 3:** Summary results for subgroup meta-analysis of the association between miR-146a rs2910164 and the risk of female neoplasms assuming homozygote (CC vs. GG), heterozygote (GC vs. GG), dominant (CC+GC vs. GG), recessive (CC vs. GC+GG), and allelic (C vs. G) models.

Models	N ^a^	Samples ^b^	OR ^b^ (95% CI) ^c^	*P* ^d^	*P*_H_^e^	I*^2^*	τ
*Ethnicity:* Asians							
Homozygote	20	5032/5371	0.89 (0.63–1.27)	0.5	<0.01	77.6 (65.8–85.3)	0.57
Heterozygote	20	5032/5371	0.93 (0.7–1.23)	0.58	<0.01	78.7 (67.7–85.9)	0.43
Dominant	20	5032/5371	0.91 (0.69–1.21)	0.51	<0.01	81.9 (73.1–87.9)	0.46
Recessive	20	5032/5371	0.94 (0.77–1.15)	0.52	<0.01	58.2 (31.4–74.5)	0.28
Allelic	20	5032/5371	0.95 (0.8–1.13)	0.57	<0.01	81.7 (72.8–87.8)	0.29
ORG^f^	20	5032/5371	0.94 (0.79–1.11)	0.48	<0.01	81.51	0.33
*Ethnicity:* Caucasians							
Homozygote	5	3859/3815	0.96 (0.39–2.35)	0.9	<0.01	85.4 (67.9–93.4)	0.61
Heterozygote	5	3859/3815	0.94 (0.75–1.19)	0.5	0.11	46.8 (0–80.5)	0.11
Dominant	5	3859/3815	0.93 (0.66–1.31)	0.58	<0.01	74.1 (35.8–89.6)	0.2
Recessive	5	3859/3815	0.99 (0.44–2.22)	0.98	<0.01	83.1 (61.4–92.6)	0.55
Allelic	5	3859/3815	0.94 (0.65–1.35)	0.66	<0.01	84.9 (66.3–93.2)	0.23
ORG	5	3859/3815	0.92 (0.74–1.16)	0.52	<0.01	80.78	0.22
*Ethnicity:* Africans							
Homozygote	2	1740/2078	0.9 (0.77–1.05)	0.07	0.9	0	0
Heterozygote	2	1740/2078	0.99 (0.89–1.11)	0.55	0.91	0	0
Dominant	2	1740/2078	0.97 (0.84–1.11)	0.2	0.87	0	0
Recessive	2	1740/2078	0.9 (0.81–1.01)	0.05	0.92	0	0
Allelic	2	1740/2078	0.96 (0.86–1.06)	0.12	0.86	0	0
ORG	2	1740/2078	0.95 (0.85–1.05)	0.35	0.84	0	0
*Breast Cancer*							
Homozygote	20	9458/10422	1.1 (0.85–1.43)	0.45	<0.01	68.7 (50.3–80.2)	0.36
Heterozygote	20	9458/10422	1.03 (0.89–1.2)	0.66	<0.01	62.6 (39.4–76.9)	0.2
Dominant	20	9458/10422	1.04 (0.88–1.22)	0.62	<0.01	70.3 (53.1–81.1)	0.22
Recessive	20	9458/10422	1.06 (0.87–1.3)	0.54	<0.01	60.8 (36.2–75.9)	0.26
Allelic	20	9458/10422	1.03 (0.91–1.17)	0.62	<0.01	74.1 (59.8–83.3)	0.18
ORG	20	9458/10422	1.03 (0.92–1.15)	0.58	<0.01	72.68	0.19
*Breast Cancer*: in Caucasians							
Homozygote	4	3758/3660	0.92 (0.26–3.23)	0.85	<0.01	89 (74.7–95.3)	0.64
Heterozygote	4	3758/3660	0.94 (0.68–1.3)	0.58	0.06	59.9 (0–86.6)	0.13
Dominant	4	3758/3660	0.92 (0.57–1.5)	0.64	<0.01	80.6 (48.9–92.6)	0.21
Recessive	4	3758/3660	0.96 (0.31–2.94)	0.91	<0.01	87.2 (69.5–94.7)	0.58
Allelic	4	3758/3660	0.93 (0.56–1.56)	0.69	<0.01	88.7 (73.5–95.1)	0.24
ORG	4	3758/3660	0.92 (0.71–1.19)	0.53	<0.01	85.58	0.23
*Breast Cancer*: in Asians							
Homozygote	13	3520/3636	1.22 (0.87–1.72)	0.22	<0.01	59.2 (24.9–77.9)	0.39
Heterozygote	13	3520/3636	1.12 (0.86–1.45)	0.37	<0.01	69.6 (46.2–82.8)	0.33
Dominant	13	3520/3636	1.13 (0.87–1.46)	0.33	<0.01	72.7 (52.4–84.3)	0.33
Recessive	13	3520/3636	1.11 (0.87–1.41)	0.36	0.04	45.5 (0–71.4)	0.23
Allelic	13	3520/3636	1.09 (0.91–1.29)	0.32	<0.01	72.1 (51.2–84)	0.22
ORG	13	3520/3636	1.09 (0.91–1.31)	0.30	<0.01	73.42	0.26
Gynecological Cancers							
Homozygote	7	1512/1735	0.55 (0.27–1.11)	0.08	<0.01	76.7 (51.2–88.9)	0.55
Heterozygote	7	1512/1735	0.62 (0.32–1.21)	0.13	<0.01	82.5 (65.3–91.2)	0.55
Dominant	7	1512/1735	0.59 (0.31–1.13)	0.09	<0.01	84 (68.7–91.8)	0.55
Recessive	7	1512/1735	0.73 (0.54–0.99)	0.04	0.11	42.3 (0–75.7)	0.19
Allelic	7	1512/1735	0.73 (0.51–1.05)	0.08	<0.01	79.8 (58.7–90.1)	0.3
ORG	7	1286/1426	0.71 (0.54–0.93)	0.01	<0.01	78.16	0.31
*High quality studies (score ≥ 9)*							
Homozygote	16	9145/10396	0.96 (0.69–1.33)	0.78	<0.01	79.6 (67.7–87.2)	0.42
Heterozygote	16	9145/10396	0.97 (0.82–1.15)	0.68	<0.01	64.4 (39.4–79.1)	0.19
Dominant	16	9145/10396	0.95 (0.77–1.16)	0.57	<0.01	76.1 (61.3–85.2)	0.24
Recessive	16	9145/10396	0.95 (0.76–1.2)	0.66	<0.01	71.7 (53.1–82.9)	0.28
Allelic	16	9145/10396	0.95 (0.82–1.1)	0.48	<0.01	81.2 (70.4–88)	0.19
ORG	16	9145/10396	0.93 (0.82–1.06)	0.32	<0.01	79.73	0.21
*Low quality studies (score < 9)*							
Homozygote	12	1927/1916	0.85 (0.51–1.41)	0.5	<0.01	73.5 (52.9–85.1)	0.72
Heterozygote	12	1927/1916	0.87 (0.57–1.33)	0.49	<0.01	79.8 (65.5–88.2)	0.51
Dominant	12	1927/1916	0.88 (0.58–1.33)	0.5	<0.01	81.1 (67.9–88.8)	0.51
Recessive	12	1927/1916	0.99 (0.72–1.38)	0.97	0.02	51.2 (2.9–75.5)	0.36
Allelic	12	1927/1916	0.97 (0.75–1.25)	0.79	<0.01	78.9 (63.7–87.7)	0.36
ORG	12	1927/1916	0.95 (0.74–1.23)	0.74	<0.01	78.23	0.39
Studies compatible with HWE							
ORG	22	9926/11189	0.90 (0.79–1.02)	0.10	<0.01	80.91	0.25

**a**: number of studies; **b:** number of samples (cases/controls)**; c:** Pooled OR and 95% CI (Random-effect model); **d:** *Pvalue* of the Z-test; **e**: *P-value* of the Q-test; **f**: ORG stands for the generalized odds ratio. For more details, refer to Section 3.4 or ref. [94,95,96].

**Table 4:** Summary results for subgroup meta-analysis of the association between miR-196a2 rs11614913 and the risk of female neoplasms assuming homozygote (TT vs. CC), heterozygote (CT vs. CC), dominant (CT+TT vs. CC), recessive (TT vs. CC+CT), and allelic model (T vs. C).

Models	N ^a^	Samples ^b^	OR ^b^ (95% CI) ^c^	*P* ^d^	*P*_Het_^e^	I*^2^*	τ
*Ethnicity*: Asians							
Homozygote	23	5815/6151	0.77 (0.6–0.97)	0.03	<0.01	62.9 (41.9–76.3)	0.38
Heterozygote	23	5815/6151	0.94 (0.8–1.09)	0.39	<0.01	47.6 (14.7–67.8)	0.22
Dominant	23	5815/6151	0.88 (0.73–1.05)	0.14	<0.01	61.9 (40.2–75.8)	0.28
Recessive	23	5815/6151	0.82 (0.67–0.98)	0.03	<0.01	62 (40.3–75.8)	0.28
Allelic	23	5815/6151	0.91 (0.83–1)	0.06	<0.01	55.1 (28.2–71.9)	0.15
ORG^f^	23	5815/6151	0.87 (0.77–0.98)	0.02	<0.01	68.30	0.23
*Ethnicity*: Caucasians							
Homozygote	5	3059/3885	0.92 (0.53–1.59)	0.69	<0.01	73.9 (35.3–89.5)	0.34
Heterozygote	5	3059/3885	0.97 (0.87–1.08)	0.44	0.7	0 (0–62.1)	0
Dominant	5	3059/3885	0.95 (0.76–1.19)	0.58	0.17	37.4 (0–76.7)	0.11
Recessive	5	3059/3885	0.95 (0.59–1.51)	0.75	<0.01	70.9 (26–88.5)	0.28
Allelic	5	3059/3885	0.97 (0.76–1.23)	0.73	0.01	70.3 (24.4–88.4)	0.15
ORG	5	3059/3885	0.96 (0.80–1.16)	0.67	0.01	68.51	0.16
Breast Cancer							
Homozygote	24	9420/11232	0.88 (0.73–1.04)	0.13	<0.01	53.3 (25.7–70.6)	0.26
Heterozygote	24	9420/11232	0.97 (0.85–1.11)	0.68	<0.01	48.9 (18–68.2)	0.16
Dominant	24	9420/11232	0.95 (0.83–1.09)	0.43	<0.01	57.2 (32.6–72.8)	0.18
Recessive	24	9420/11232	0.9 (0.78–1.04)	0.15	<0.01	50.6 (20.9–69.1)	0.21
Allelic	24	9420/11232	0.95 (0.87–1.04)	0.28	<0.01	61.8 (40.5–75.4)	0.14
ORG	24	9646/11541	0.94 (0.86–1.04)	0.26	<0.01	61.66	0.16
*Breast Cancer*: among Asians							
Homozygote	16	4281/4428	0.82 (0.65–1.02)	0.07	0.04	41 (0–67.4)	0.23
Heterozygote	16	4281/4428	0.93 (0.77–1.12)	0.42	0.02	48.7 (8.5–71.2)	0.2
Dominant	16	4281/4428	0.9 (0.74–1.09)	0.24	<0.01	55.6 (22.1–74.7)	0.23
Recessive	16	4281/4428	0.89 (0.72–1.09)	0.23	0.01	50.6 (12.3–72.2)	0.21
Allelic	16	4281/4428	0.92 (0.81–1.04)	0.18	<0.01	59.5 (29.8–76.6)	0.15
ORG	16	4507/4737	0.91 (0.79–1.04)	0.19	<0.01	60.65	0.19
*Breast Cancer*: among Caucasians							
Homozygote	5	2979/3885	0.93 (0.53–1.63)	0.74	<0.01	74.3 (36.3–89.6)	0.35
Heterozygote	5	2979/3885	0.97 (0.87–1.09)	0.53	0.7	0 (0–62)	0
Dominant	5	2979/3885	0.96 (0.76–1.22)	0.68	0.16	38.9 (0–77.4)	0.11
Recessive	5	2979/3885	0.95 (0.59–1.52)	0.77	<0.01	71 (26.4–88.6)	0.29
Allelic	5	2979/3885	0.98 (0.76–1.25)	0.81	<0.01	70.9 (26.1–88.5)	0.15
ORG	5	2979/3885	0.97 (0.80–1.17)	0.76	0.1	69.08	0.17
Gynecological Cancers							
Homozygote	8	1614/1928	0.69 (0.38–1.26)	0.19	<0.01	78.3 (57.4–89)	0.62
Heterozygote	8	1614/1928	0.93 (0.66–1.29)	0.6	0.06	47.9 (0–76.8)	0.3
Dominant	8	1614/1928	0.83 (0.55–1.26)	0.33	<0.01	70 (37.6–85.6)	0.42
Recessive	8	1614/1928	0.71 (0.47–1.09)	0.1	<0.01	71.9 (42.3–86.3)	0.4
Allelic	8	1614/1928	0.89 (0.75–1.05)	0.13	0.11	40.3 (0–73.6)	0.15
ORG	8	1614/1928	0.78 (0.61–0.99)	0.04	<0.01	74.97	0.30
*Gynecological Cancers*: among Asians							
Homozygote	7	1534/1723	0.68 (0.33–1.37)	0.23	<0.01	81.2 (62.1–90.7)	0.6
Heterozygote	7	1534/1723	0.96 (0.65–1.41)	0.8	0.047	52.8 (0–79.9)	0.27
Dominant	7	1534/1723	0.84 (0.51–1.39)	0.44	<0.01	74.2 (45–87.9)	0.41
Recessive	7	1534/1723	0.69 (0.43–1.12)	0.11	<0.01	75.1 (47.1–88.2)	0.38
Allelic	7	1534/1723	0.89 (0.74–1.09)	0.21	0.06	48.8 (0–78.3)	0.14
ORG	7	1534/1723	0.77 (0.59–1.02)	0.06	<0.01	78.46	0.32
*Gynecological Cancers*: OCa							
Homozygote	5	913/1305	0.77 (0.35–1.69)	0.41	0.02	64.7 (7.3–86.5)	0.42
Heterozygote	5	913/1305	0.94 (0.48–1.85)	0.82	0.05	58.5 (0–84.6)	0.32
Dominant	5	913/1305	0.87 (0.44–1.74)	0.62	0.03	63.5 (3.6–86.2)	0.34
Recessive	5	913/1305	0.73 (0.54–1)	0.05	0.32	14.5 (0–82.2)	0.1
Allelic	5	913/1305	0.85 (0.63–1.17)	0.23	0.06	56.7 (0–84)	0.17
ORG	5	913/1305	0.82 (0.64–1.05)	0.12	0.05	57.01	0.20
*High quality studies (score ≥ 9)*							
Homozygote	17	8689/10682	0.79 (0.67–0.93)	<0.01	0.01	48 (8.7–70.4)	0.2
Heterozygote	17	8689/10682	0.96 (0.88–1.04)	0.26	0.35	8.8 (0–45.2)	0.05
Dominant	17	8689/10682	0.9 (0.81–0.99)	0.049	0.04	41.8 (0–67.2)	0.12
Recessive	17	8689/10682	0.83 (0.73–0.93)	<0.01	0.08	33.9 (0–63.2)	0.12
Allelic	17	8689/10682	0.9 (0.83–0.98)	0.01	<0.01	56.5 (25.3–74.7)	0.11
ORG	17	8689/10682	0.88 (0.81–0.96)	<0.01	<0.01	57.12	0.12
*Low quality studies (score < 9)*							
Homozygote	14	2345/2273	0.89 (0.58–1.37)	0.57	<0.01	76 (59.7–85.7)	0.67
Heterozygote	14	2345/2273	1.04 (0.75–1.44)	0.78	<0.01	67.6 (43.4–81.5)	0.41
Dominant	14	2345/2273	0.99 (0.7–1.39)	0.94	<0.01	75.2 (58.3–85.3)	0.47
Recessive	14	2345/2273	0.9 (0.63–1.28)	0.53	<0.01	74.9 (57.6–85.1)	0.5
Allelic	14	2345/2273	1.03 (0.88–1.2)	0.71	<0.01	59.9 (27.9–77.7)	0.21
ORG	14	2345/2273	0.96 (0.77–1.21)	0.78	<0.01	77.76	0.37
Studies compatible with HWE							
ORG	22	9917/11720	0.89 (0.82–0.98)	0.02	<0.01	64.15	0.15

**a**: number of studies; **b**: number of samples (cases/controls); **c**: Pooled OR and 95% CI (Random-effect model); **d**: *Pvalue* of the Z-test; **e**: *P-value* of the Q-test;

**Table 5:** Summary results for subgroup meta-analysis of the association between miR-27a rs895819 and the risk of female neoplasms assuming homozygote (GG vs. AA), heterozygote (AG vs. AA), dominant (AG+GG vs. AA), recessive (GG vs. AA+AG), and allelic model (G vs. A).

Models	N ^a^	Samples ^b^	OR ^b^ (95% CI) ^c^	*P* ^d^	*P*_Het_^e^	I*^2^*	τ
*Ethnicity*: Asians							
Homozygote	8	2165/2429	0.9 (0.53–1.5)	0.63	<0.01	76.1 (52.2–88)	0.53
Heterozygote	8	2165/2429	0.93 (0.69–1.24)	0.57	<0.01	67.2 (30.7–84.4)	0.27
Dominant	8	2165/2429	0.91 (0.68–1.22)	0.47	<0.01	71.6 (41.4–86.2)	0.28
Recessive	8	2165/2429	0.89 (0.56–1.44)	0.6	<0.01	74.7 (49–87.5)	0.47
Allelic	8	2165/2429	0.91 (0.72–1.16)	0.38	<0.01	76.9 (54–88.4)	0.24
ORG^f^	8	2165/2429	0.89 (0.72–1.09)	0.26	<0.01	73.73	0.25
*Ethnicity*: Caucasians							
Homozygote	3	2481/3197	0.85 (0.65–1.1)	0.11	0.66	0 (0–75.4)	0
Heterozygote	3	2481/3197	0.84 (0.67–1.05)	0.08	0.43	0 (0–87.7)	0
Dominant	3	2481/3197	0.84 (0.71–0.99)	0.04	0.61	0 (0–79.1)	0
Recessive	3	2481/3197	0.92 (0.66–1.29)	0.41	0.48	0 (0–85.7)	0
Allelic	3	2481/3197	0.89 (0.83–0.95)	0.02	0.86	0 (0–28.5)	0
ORG	3	2481/3197	0.86 (0.78–0.95)	<0.01	0.79	0	0
Breast Cancer							
Homozygote	12	6556/8044	0.8 (0.65–0.99)	0.04	0.02	50.1 (3.2–74.3)	0.2
Heterozygote	12	6556/8044	0.9 (0.77–1.05)	0.17	<0.01	56.8 (17.8–77.3)	0.15
Dominant	12	6556/8044	0.87 (0.76–1.01)	0.07	<0.01	56.2 (16.4–77)	0.14
Recessive	12	6556/8044	0.83 (0.68–1)	0.05	0.02	51.9 (7.2–75.1)	0.19
Allelic	12	6556/8044	0.88 (0.79–0.98)	0.03	<0.01	59.1 (22.8–78.4)	0.11
ORG	12	6556/8044	0.86 (0.78–0.95)	<0.01	<0.01	57.55	0.12
*Breast Cancer*: in Asians							
Homozygote	7	2062/2012	0.76 (0.5–1.17)	0.17	0.01	63.6 (17.7–83.9)	0.38
Heterozygote	7	2062/2012	0.91 (0.65–1.28)	0.53	<0.01	70.9 (36.6–86.7)	0.28
Dominant	7	2062/2012	0.87 (0.63–1.19)	0.32	<0.01	71.4 (37.9–86.8)	0.27
Recessive	7	2062/2012	0.76 (0.53–1.11)	0.13	0.03	58.2 (3.4–81.9)	0.32
Allelic	7	2062/2012	0.86 (0.69–1.07)	0.14	<0.01	71 (36.7–86.7)	0.2
ORG	7	2062/2012	0.83 (0.68–1.02)	0.08	<0.01	69.57	0.22
*Breast Cancer*: in Caucasians							
Homozygote	3	2401/3197	0.85 (0.72–1.02)	0.06	0.83	0 (0–42.4)	0
Heterozygote	3	2401/3197	0.84 (0.67–1.05)	0.08	0.43	0 (0–87.6)	0
Dominant	3	2401/3197	0.84 (0.71–0.99)	0.045	0.61	0 (0–79.2)	0
Recessive	3	2401/3197	0.93 (0.72–1.21)	0.36	0.64	0 (0–76.4)	0
Allelic	3	2401/3197	0.89 (0.84–0.94)	0.01	0.92	0 (0–0)	0
ORG	3	2401/3197	0.86 (0.78–0.95)	<0.01	0.82	0	0
*High quality studies (score ≥ 9)*							
Homozygote	10	5915/7752	0.87 (0.61–1.23)	0.38	<0.01	71.2 (45–84.9)	0.31
Heterozygote	10	5915/7752	0.93 (0.81–1.07)	0.26	0.06	45.2 (0–73.7)	0.12
Dominant	10	5915/7752	0.91 (0.79–1.06)	0.2	0.03	52.6 (2.9–76.9)	0.13
Recessive	10	5915/7752	0.88 (0.63–1.22)	0.39	<0.01	72.4 (47.9–85.4)	0.29
Allelic	10	5915/7752	0.92 (0.8–1.06)	0.21	<0.01	67 (35.8–83.1)	0.12
ORG	10	5915/7752	0.90 (0.81–1.00)	0.07	<0.01	60.97	0.12
*Low quality studies (score < 9)*							
Homozygote	3	828/709	0.81 (0.36–1.78)	0.36	0.34	8.5 (0–90.5)	0.1
Heterozygote	3	828/709	0.78 (0.29–2.11)	0.4	0.01	76.3 (22.5–92.8)	0.35
Dominant	3	828/709	0.77 (0.31–1.93)	0.35	0.02	75.3 (18.3–92.5)	0.32
Recessive	3	828/709	0.87 (0.53–1.44)	0.36	0.59	0 (0–80.1)	0
Allelic	3	828/709	0.82 (0.44–1.52)	0.3	0.04	69.9 (0–91.2)	0.21
ORG	3	828/709	0.78 (0.55–1.12)	0.18	0.02	72.78	0.26
Studies compatible with HWE							
ORG	12	6303/7654	0.88 (0.78–0.99)	0.35	<0.01	65.17	0.15

**a**: number of studies; **b:** number of samples (cases/controls); **c**: Pooled OR and 95% CI (Random-effect model); **d**: *Pvalue* of the Z-test; **e**: *P-value* of the Q-test; **f**: ORG stands for the generalized odds ratio. For more details, refer to Section 3.4 or ref. [94,95,96].

**Table 6:** Summary results for subgroup meta-analysis of the association between miR-499 rs3746444 and the risk of female neoplasms under homozygote (CC vs. TT), heterozygote (TC vs. TT), dominant (TC+CC vs. TT), recessive (CC vs. TT+TC), and allelic (C vs. T) models.

Models	N ^a^	Samples ^b^	OR (95% CI) ^c^	*P* ^d^	*P*_Het_^e^	I*^2^*	τ
*Ethnicity*: *Asians*							
Homozygote	14	3908/4198	1.5 (0.98–2.3)	0.06	<0.01	68.5 (45.2–81.9)	0.50
Heterozygote	14	3908/4198	1.18 (0.93–1.5)	0.16	<0.01	64.8 (37.8–80.1)	0.27
Dominant	14	3908/4198	1.26 (0.97–1.64)	0.07	<0.01	70.8 (49.7–83.1)	0.29
Recessive	14	3908/4198	1.4 (0.97–2.02)	0.07	<0.01	65.5 (39.2–80.4)	0.43
Allelic	15	3951/4246	1.3 (1.05–1.61)	0.02	<0.01	74.2 (57.1–84.5)	0.26
ORG^f^	14	3908/4198	1.26 (1.05–1.51)	0.01	<0.01	74.91	0.29
*Breast Cancer*							
Homozygote	12	6653/8376	1.32 (0.93–1.87)	0.11	<0.01	61.6 (28.1–79.5)	0.34
Heterozygote	12	6653/8376	1.08 (0.91–1.28)	0.35	0.01	54.1 (11.9–76.1)	0.15
Dominant	12	6653/8376	1.14 (0.94–1.38)	0.17	<0.01	65.1 (35.6–81.1)	0.18
Recessive	12	6653/8376	1.28 (0.91–1.78)	0.14	<0.01	61.4 (27.6–79.4)	0.33
Allelic	13	6696/8424	1.17 (0.97–1.41)	0.09	<0.01	74.5 (55.9–85.2)	0.19
ORG	12	6653/8376	1.14 (1.00–1.30)	0.04	<0.01	69.38	0.18
*Breast Cancer: in Asians*							
Homozygote	9	2977/3133	1.43 (0.85–2.41)	0.15	0.01	62.2 (22.1–81.7)	0.41
Heterozygote	9	2977/3133	1.13 (0.85–1.51)	0.36	0.01	60.9 (18.9–81.1)	0.23
Dominant	9	2977/3133	1.21 (0.9–1.64)	0.18	<0.01	66.2 (31.5–83.3)	0.24
Recessive	9	2977/3133	1.36 (0.83–2.24)	0.19	0.01	63.2 (24.3–82.1)	0.4
Allelic	10	3020/3181	1.24 (0.96–1.62)	0.09	<0.01	72.9 (48.7–85.6)	0.24
ORG	9	2977/3133	1.21 (0.99–1.47)	0.05	<0.01	69.35	0.23
*Gynecological cancer*							
Homozygote	5	931/1065	1.65 (0.56–4.88)	0.27	<0.01	79.1 (50.3–91.2)	0.8
Heterozygote	5	931/1065	1.26 (0.66–2.4)	0.37	<0.01	72.4 (30.7–89)	0.42
Dominant	5	931/1065	1.35 (0.64–2.83)	0.32	<0.01	78.8 (49.4–91.1)	0.47
Recessive	5	931/1065	1.46 (0.64–3.35)	0.27	<0.01	74.8 (37.8–89.8)	0.59
Allelic	5	931/1065	1.42 (0.8–2.54)	0.17	<0.01	74.7 (37.5–89.8)	0.35
ORG	5	931/1065	1.34 (0.88–2.06)	0.17	<0.01	82.77	0.44
*High quality studies (score ≥ 9)*							
Homozygote	10	6433/8387	1.43 (1.1–1.85)	0.01	0.04	49.7 (0–75.6)	0.25
Heterozygote	10	6433/8387	1.12 (0.97–1.3)	0.11	0.03	50.2 (0–75.8)	0.13
Dominant	10	6433/8387	1.19 (1.03–1.36)	0.02	0.02	55.4 (9.2–78.1)	0.13
Recessive	10	6433/8387	1.39 (1.07–1.82)	0.02	0.03	51.8 (1–76.5)	0.26
Allelic	10	6433/8387	1.21 (1.06–1.39)	0.01	<0.01	68.2 (38.5–83.6)	0.14
ORG	10	6433/8387	1.20 (1.07–1.35)	<0.01	<0.01	61.00	0.13
*Low quality studies (score < 9)*							
Homozygote	7	1151/1054	1.25 (0.44–3.52)	0.62	<0.01	82.2 (64.5–91.1)	0.89
Heterozygote	7	1151/1054	1.13 (0.65–1.98)	0.6	<0.01	76.2 (50.1–88.7)	0.5
Dominant	7	1151/1054	1.18 (0.62–2.23)	0.55	<0.01	83.7 (68.1–91.7)	0.59
Recessive	7	1151/1054	1.13 (0.49–2.59)	0.74	<0.01	78.1 (54.6–89.4)	0.66
Allelic	8	1194/1102	1.22 (0.75–1.98)	0.36	<0.01	85.1 (72.5–91.9)	0.51
ORG	7	1151/1054	1.13 (0.73–1.76)	0.56	<0.01	86.53	0.54
*Studies compatible with HWE*							
ORG	13	6851/8615	1.19 (1.01–1.39)	0.03	<0.01	79.42	0.24

**a**: number of studies; **b**: number of samples (cases/controls); **c**: Pooled OR and 95% CI (Random-effect model); **d**: *Pvalue* of the Z-test; **e**: *P-value* of the Q-test; **f**: ORG stands for the generalized odds ratio. For more details, refer to Section 3.4 or ref. [94,95,96].

**Table 7:** Summary results of subgroup meta-analysis for the remaining miRNA polymorphisms. miR-423 rs6505162, miR-149 rs2292832, miR-100 rs1834306, miR-605 rs2043556, miR-608 rs4919510, miR-218 rs11134527, miR-34b/c rs4938723, and miR-124 rs531564.

Genetic Models	N ^a^	Samples ^b^	OR (95% CI) ^c^	*P* ^d^	*P*_H_^e^	I*^2^*	τ
*miR-423 rs6505162: HWE compatible studies*							
Homozygote(AA vs. CC)	8	3405/4149	1.04 (0.73–1.47)	0.8	0.06	47.9 (0–76.8)	0.26
Heterozygote(AC vs. CC)	8	3405/4149	0.97 (0.7–1.36)	0.86	0	69.5 (36.3–85.3)	0.29
Dominant(AC+AA vs. CC)	8	3405/4149	0.97 (0.7–1.34)	0.82	0	70.9 (39.8–85.9)	0.29
Recessive(AA vs. CC+AC)	8	3405/4149	0.97 (0.86–1.08)	0.5	0.63	0 (0–56.4)	0
Allelic(A vs. C)	8	3405/4149	0.96 (0.8–1.16)	0.66	0.01	61.7 (17.1–82.3)	0.15
ORG^f^	8	3405/4149	0.94 (0.80–1.12)	0.54	<0.01	64.58	0.18
*miR-423 rs6505162: BCa*							
Homozygote(AA vs. CC)	9	3426/4273	1.17 (0.72–1.89)	0.48	<0.01	68.8 (37.5–84.4)	0.43
Heterozygote(AC vs. CC)	9	3426/4273	1.17 (0.68–2.01)	0.52	<0.01	84.2 (71.6–91.2)	0.47
Dominant(AC+AA vs. CC)	9	3426/4273	1.15 (0.69–1.92)	0.55	<0.01	85 (73.4–91.6)	0.46
Recessive(AA vs. CC+AC)	9	3426/4273	0.99 (0.82–1.2)	0.94	0.29	17 (0–59)	0.1
Allelic(A vs. C)	9	3426/4273	1.05 (0.76–1.44)	0.74	<0.01	82.5 (68.2–90.4)	0.27
ORG	9	3426/4273	1.04 (0.82–1.31)	0.72	<0.01	82.30	0.30
*miR-423 rs6505162 in Asians*							
Homozygote(AA vs. CC)	5	962/1068	1.2 (0.43–3.29)	0.65	<0.01	72.2 (30.2–89)	0.76
Heterozygote(AC vs. CC)	5	962/1068	1.14 (0.34–3.84)	0.78	<0.01	90.3 (80.2–95.2)	0.71
Dominant(AC+AA vs. CC)	5	962/1068	1.12 (0.37–3.41)	0.79	<0.01	90.4 (80.5–95.3)	0.68
Recessive(AA vs. CC+AC)	5	962/1068	1.04 (0.58–1.87)	0.85	0.33	14 (0–82.1)	0.18
Allelic(A vs. C)	5	962/1068	1.06 (0.51–2.18)	0.85	<0.01	88.1 (74.8–94.4)	0.49
ORG	5	962/1068	1.04 (0.62–1.74)	0.87	<0.01	87.60	0.54
*miR-149 rs2292832*: *HWE compatible studies*							
Homozygote(CC vs. TT)	4	1859/2114	0.79 (0.48–1.3)	0.23	0.11	49.6 (0–83.3)	0.24
Heterozygote(CT vs. TT)	4	1859/2114	0.87 (0.68–1.11)	0.17	0.5	0 (0–80.5)	0
Dominant(CT+CC vs. TT)	4	1859/2114	0.84 (0.64–1.1)	0.14	0.37	4.4 (0–85.4)	0.04
Recessive(CC vs. TT+CT)	4	1859/2114	0.87 (0.57–1.32)	0.35	0.1	51.2 (0–83.9)	0.19
Allelic(C vs. T)	4	1859/2114	0.89 (0.7–1.12)	0.2	0.08	55.9 (0–85.4)	0.12
ORG	4	1859/2114	0.87 (0.72–1.04)	0.13	0.09	52.24	0.12
*miR-100 rs1834306*: *HWE compatible studies*							
Homozygote(AA vs. GG)	3	903/1190	0.99 (0.62–1.6)	0.95	0.47	0 (0–86.2)	0
Heterozygote(AG vs. GG)	3	903/1190	0.9 (0.49–1.65)	0.54	0.24	30.4 (0–92.8)	0.14
Dominant(AA+AG vs. GG)	3	903/1190	0.95 (0.64–1.39)	0.6	0.45	0 (0–87.1)	0
Recessive(AA vs. AG+GG)	5	1705/1911	0.95 (0.69–1.32)	0.71	0.12	45.5 (0–80)	0.16
Allelic(A vs. G)	3	903/1190	0.99 (0.78–1.25)	0.86	0.5	0 (0–85.1)	0
ORG	3	903/1190	0.98 (0.84–1.14)	0.85	0.52	0	0
*miR-605 rs2043556 in Asians*							
Homozygote(GG vs. AA)	3	609/728	0.64 (0.01–31.22)	0.67	<0.01	90.8 (75.9–96.5)	1.39
Heterozygote(GA vs. AA)	3	609/728	0.94 (0.68–1.3)	0.5	0.72	0 (0–68)	0
Dominant(GA+GG vs. AA)	3	609/728	0.89 (0.47–1.68)	0.51	0.27	23.4 (0–92)	0.12
Recessive(GG vs. AA+GA)	3	609/728	0.64 (0.01–33.78)	0.68	<0.01	92 (79.7–96.8)	1.4
Allelic(G vs. A)	3	609/728	0.83 (0.26–2.69)	0.57	<0.01	87.6 (65.1–95.6)	0.39
ORG	3	609/728	0.79 (0.46–1.36)	0.40	<0.01	84.24	0.43
*miR-608 rs4919510 in Asians*							
Homozygote(GG vs. CC)	4	1675/2382	1.16 (0.96–1.41)	0.11	0.98	0 (0–0)	0
Heterozygote(GC vs. CC)	4	1675/2382	1.08 (0.55–2.13)	0.75	0.02	68.4 (8.4–89.1)	0.27
Dominant(GG+GC vs. CC)	4	1675/2382	1.06 (0.57–1.99)	0.78	0.03	66.1 (0.5–88.4)	0.24
Recessive(GG vs. GC+CC)	4	1675/2382	1.01 (0.87–1.17)	0.85	0.91	0 (0–12.7)	0
Allelic(G vs. C)	4	1675/2382	1.02 (0.73–1.42)	0.86	0.1	51.4 (0–83.9)	0.11
ORG	4	1675/2382	1.00 (0.83–1.22)	0.93	0.08	55.24	0.14
*miR-218 rs11134527: CC*							
Homozygote(AA vs. GG)	3	2869/2687	1.08 (0.47–2.5)	0.73	0.02	75.8 (20.3–92.6)	0.26
Heterozygote(AG vs. GG)	3	2869/2687	1.09 (0.6–1.96)	0.6	0.09	59.1 (0–88.3)	0.17
Dominant(AA+AG vs. GG)	3	2869/2687	1.08 (0.54–2.16)	0.68	0.03	71.2 (2.2–91.5)	0.22
Recessive(AA vs. AG+GG)	3	2869/2687	1.03 (0.68–1.56)	0.82	0.09	58.2 (0–88.1)	0.12
Allelic(A vs. G)	3	2869/2687	1.03 (0.7–1.52)	0.74	0.02	74.7 (15.8–92.4)	0.12
ORG	3	2869/2687	1.03 (0.86–1.24)	0.69	0.02	72.87	0.13
*miR-34b/c rs4938723: BCa*							
Homozygote(CC vs. TT)	3	2208/1967	0.9 (0.58–1.4)	0.4	0.39	0 (0–89.1)	0
Heterozygote(CT vs. TT)	3	2208/1967	1.02 (0.73–1.42)	0.82	0.29	19.5 (0–91.6)	0.06
Dominant(CT+CC vs. TT)	3	2208/1967	1 (0.74–1.36)	0.99	0.29	19.9 (0–91.7)	0.06
Recessive(CC vs. TT+CT)	3	2208/1967	0.89 (0.59–1.35)	0.35	0.39	0 (0–89)	0
Allelic(C vs. T)	3	2208/1967	0.98 (0.78–1.22)	0.7	0.3	16.1 (0–91.3)	0.04
ORG	3	2208/1967	1.01 (0.89–1.15)	0.77	0.27	22.79	0.05
*miR-124 rs531564: high quality studies*							
Homozygote(GG vs. CC)	3	1055/1052	0.44 (0.28–0.68)	0.01	0.92	0 (0–0)	0
Heterozygote(GC vs. CC)	3	1055/1052	1.04 (0.37–2.86)	0.88	0.2	36.9 (0–80.0)	0.05
Dominant(GG+GC vs. CC)	3	1055/1052	0.93 (0.27–3.13)	0.82	0.12	52.5 (0–86.4)	0.09
Recessive(GG vs. GC+CC)	3	1055/1052	0.74 (0.57–0.96)	0.02	0.73	0 (0–65.7)	0
Allelic(G vs. C)	3	1055/1052	0.88 (0.52–1.51)	0.44	0.20	36.0 (0–79.5)	0.02
ORG	3	1055/1052	1.07 (0.80–1.44)	0.63	0.17	43.47	0.17

**a**: number of studies; **b**: number of samples (cases/controls); **c**: Pooled OR and 95% CI (Random-effect model); **d**: *Pvalue* of the Z-test; **e**: *P-value* of the Q-test; **f**: ORG stands for the generalized odds ratio. For more details, refer to Section 3.4 or ref. [94,95,96].

## Abbreviations

BCa	Breast cancer
CCa	Cervical cancer
CI	Confidence interval
ECa	Endometrial Cancer
GCa	Gynecological cancers
HWE	Hardy-Weinberg equilibrium
OR	Odds ratio
ORG	Generalized odds ratio
OCa	Ovarian cancer
SNP	Single nucleotide polymorphism
